# In Situ Profiling of Nanoscale Strains Uncovers Mechano‐Architectural Predictors of Aging and Osteoarthritis Emergence

**DOI:** 10.1002/advs.76716

**Published:** 2026-07-23

**Authors:** Aikta Sharma, Lucinda A. E. Evans, Lucie E. Bourne, Jishizhan Chen, Alissa L. Parmenter, Joseph Brunet, Kamel Madi, Sebastian Marussi, Andrew A. Pitsillides, Peter D. Lee, Katherine A. Staines

**Affiliations:** ^1^ Department of Mechanical Engineering University College London London UK; ^2^ Research Complex at Harwell Rutherford Appleton Laboratory Didcot UK; ^3^ Centre for Lifelong Health School of Applied Sciences University of Brighton Brighton UK; ^4^ Comparative Biomedical Sciences Royal Veterinary College London UK; ^5^ 3Dmagination Ltd Rutherford Appleton Laboratory Didcot UK

**Keywords:** bone, cartilage, digital volume correlation, growth plate, in situ synchrotron computed tomography, osteoarthritis

## Abstract

Coordinated load transfer across knee joint compartments underpins lifelong joint function, yet dysregulated mechanics are also widely implicated in the etiology of osteoarthritis (OA). How physiological loads are accommodated in the healthy joint and how regionalized architectural alterations reconfigure joint‐level mechanics to promote OA, however, remain unresolved. Here, we integrate in situ mechanical loading of murine tibial epiphyses with phase‐contrast synchrotron X‐ray computed tomography and digital volume correlation to quantify three‐dimensional, compartment‐specific load‐bearing behavior in intact healthy (CBA) and OA‐prone (STR/Ort) knee joints. We find that raised focal strain concentrations emerge within the subchondral plate and precede histological cartilage degeneration in STR/Ort joints at 10 weeks of age. In contrast, these strain concentrations are absent in both young and aging CBA mice, where mechanical strain is preferentially transmitted to locations distant from the articular surface. Finite element modeling further reveals that strain localization in STR/Ort joints is governed by region‐specific microstructural incongruities. Together, these findings demonstrate that epiphyseal microarchitecture preserves mechanical homeostasis during healthy aging, whereas spatial disorganization of subchondral microarchitecture renders the epiphysis susceptible to load‐induced failure. Collectively, this work identifies mechano‐architectural misalignment within the mineralized phase of the tibial epiphysis as an early mechanical promoter of OA emergence.

## Graphical abstract

In situ mechanical loading of osteoarthritis‐prone murine knee joints reveals that spatial alterations in subchondral plate microarchitecture precede cartilage loss and promote a shift from coordinated epiphyseal strain dissipation in healthy joints to focal subchondral strain concentration in osteoarthritis‐prone joints. This study identifies microstructural misalignment as an early mechanical determinant of osteoarthritis development.

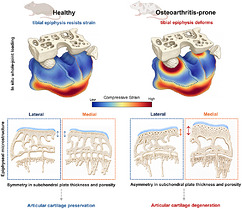

## 1 Introduction

Functional integrity across the skeletal system is dependent upon the precise alignment of tissue architecture with prevailing load‐bearing demands. At all hierarchical levels, microstructural organization governs tissue compliance under load and dictates how mechanical stresses are distributed and transmitted across adjacent tissue types [1, 2]. In diarthrodial joints, this structural‐mechanical coupling is particularly critical, as sustained joint performance throughout life relies on tightly regulated interactions between anatomically distinct yet mechanically complementary compartments. Disruption of this mechano‐architectural balance is widely acknowledged as a primary driver of joint failure in age‐related osteoarthritis (OA), the most prevalent degenerative joint disease worldwide [3, 4]. However, the mechanisms by which physiological loads are accommodated within the intact joint during healthy aging, and how early mechano‐architectural perturbations across joint compartments alter joint‐level mechanics in the initial stages of OA remain poorly defined. Thus, despite extensive investigation, predictive biomarkers and disease‐modifying therapies for OA remain elusive [5, 6]. This persistent translational gap reflects both the complexity of the mechanical and structural interactions between knee joint tissues, and our limited ability to resolve how their integrated mechanical behavior governs joint function during healthy aging and disease onset.

Within the knee, the tibial epiphysis is central to joint integrity and load‐bearing capacity. The epiphysis comprises an integrated osteochondral system in which articular cartilage, the subchondral plate (SCP), and epiphyseal trabecular bone retain distinct physiology and material properties [7, 8, 9, 10], yet operate as a mechanically continuous unit [11]. Articular cartilage is a highly specialized, avascular connective tissue that enables low friction at articulating surfaces [12] while resisting compressive stress generated during loading [7]. Beneath this, the SCP forms a key biomechanical interface that supports the articular cartilage and transmits intra‐articular loads toward the underlying epiphyseal trabecular and adjacent cortical bone compartments [13, 14, 15, 16]. Progressive articular cartilage degeneration in OA is either accompanied by, or perhaps preceded by, thickening and stiffening of the SCP [17, 18], a process thought to disrupt mechanical continuity across the osteochondral interface, promoting maladaptive subchondral remodeling and compromising articular cartilage protection [19, 20]. Whether OA arises from an intrinsic mismatch between the joint's compartmental architecture and load‐bearing behavior, however, remains unresolved, thus limiting our ability to identify early mechanical biomarkers of disease susceptibility. Bridging this critical gap requires imaging approaches capable of simultaneously resolving tissue microstructure across the whole joint organ while subjected to physiologically relevant loads.

Previous studies have combined mechanical loading with micro‐computed tomography (µCT) to investigate osteochondral mechanics in OA, but only in isolated osteochondral plugs [21, 22, 23] in which native intercompartmental interactions are disrupted, or on excised human joints with end‐stage disease [24, 25] precluding insight into the emergence of early pathological changes. Phase‐contrast synchrotron X‐ray computed tomography (sCT) offers a powerful solution to these limitations. We previously established sCT as a high‐resolution modality capable of visualizing intact murine joints across hierarchical scales under habitual weight‐bearing load, offering superior contrast, speed and spatial resolution compared to laboratory‐based µCT [26]. Indeed, sCT has been widely applied to characterize joint tissue microstructure in several animal models [27, 28, 29, 30, 31] and human joints [28, 32, 33, 34, 35, 36], yet the direct assessment of mechanical behavior has remained elusive. Digital volume correlation (DVC) overcomes this limitation by tracking voxel‐level grayscale patterns across sequential volumetric images acquired under load [37, 38], enabling full‐field, three‐dimensional (3D) strain quantification when integrated with CT‐based imaging modalities [21, 39, 40, 41, 42, 43, 44]. Furthermore, when combined with strain‐calibrated finite element (FE) modeling, these methods enable inference of local load‐bearing behavior and tissue‐level mechanical competence [25, 39, 45, 46, 47].

**FIGURE 1:**
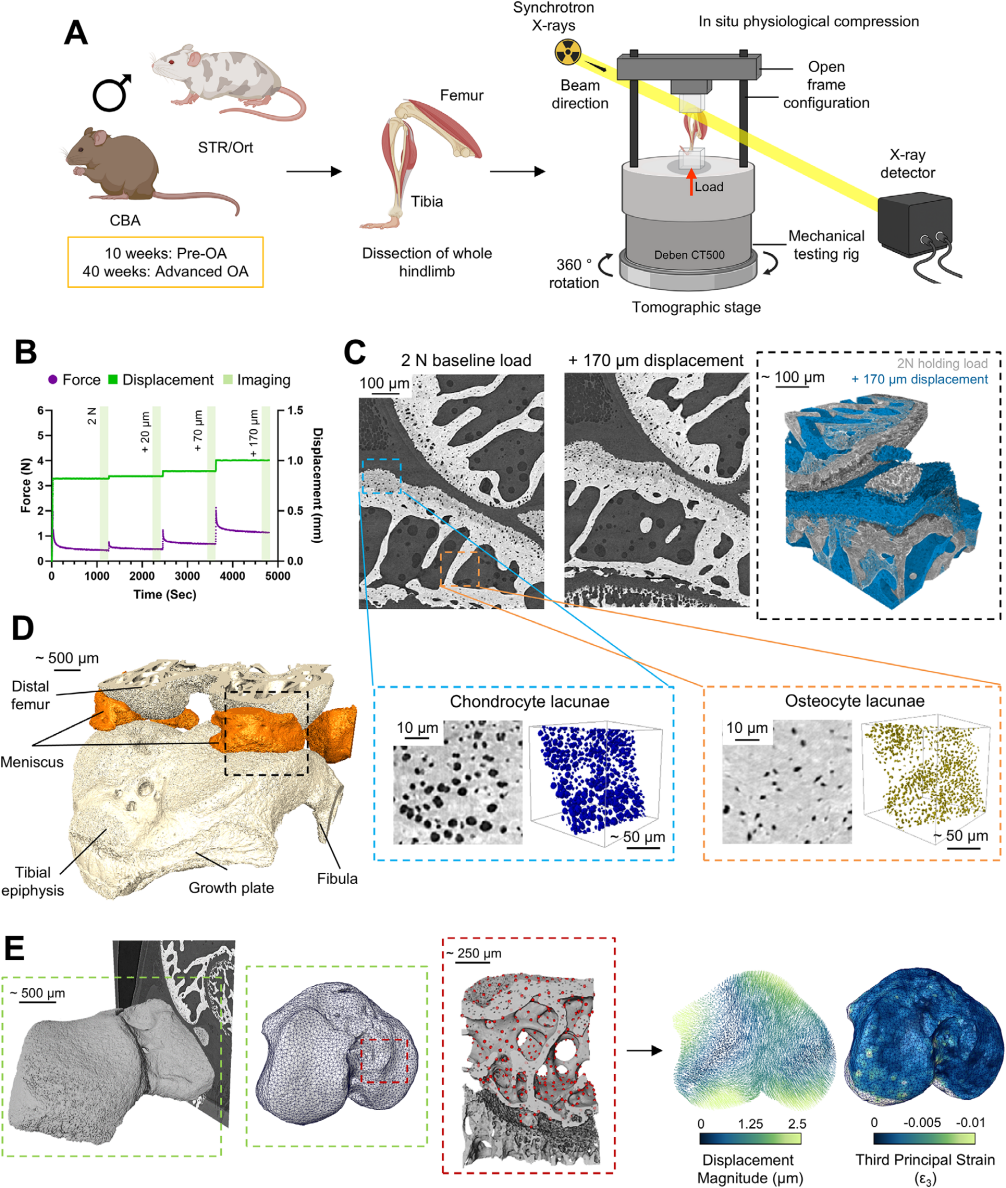
Characterization of load‐induced compressive strain in the epiphyses of healthy CBA and OA‐prone STR/Ort knee joints with phase‐contrast sCT and DVC. Schematic of methodology showing isolation of hindlimbs from 10‐ and 40‐week‐old male CBA and STR/Ort mice and mounting in a Deben CT500 compression stage with a bespoke open frame for in situ mechanical testing and sCT (A). Representative force (purple line) and displacement (green line) readings showing acquisition of sCT images (green rectangle) after application of a 2 N baseline load and three displacement‐controlled compression steps of 20, 70 and 170 µm (B). Sagittal sCT images of the medial tibial epiphysis following application of the 2 N baseline load (C, left) and in response to 170 µm compressive displacement (C, middle) are shown in 3D (C, right; gray, 2 N baseline load; blue, 170 µm displacement; scale bar = 100 µm). Zoomed sCT images (scale bar = 100 µm) and 3D renders of epiphyseal microstructure (scale bar = 10 µm) show chondrocyte lacunae (blue box) and osteocyte lacunae (orange box) within the epiphyseal subchondral regions. 3D rendering of the CBA joint with anatomical regions labeled (D; scale bar = 500 µm). Tibial epiphyses and growth plate bridges were segmented using a region‐growing algorithm, followed by closure of chondrocyte and osteocyte lacunae (E, left; scale bar = 500 µm). The resulting structures were used to generate FE tetrahedral meshes (E, green hatched box). Corresponding point cloud nodes (red dots) overlaid onto the epiphyseal microstructure (E, red hatched box; scale bar = 250 µm) were subsequently used to calculate displacement and strain magnitude by DVC (E, right). Schematic shown in A was created using BioRender.com.

To determine whether compartment‐specific architectural differences alter load‐transfer across the intact murine tibial epiphysis, we integrated phase‐contrast sCT with DVC to resolve full‐field, 3D strain distributions in intact mouse knee joints under physiological compression. Enabled by the Extremely Brilliant Source upgrade at the European Synchrotron Radiation Facility, this approach permits high‐resolution in situ strain mapping across the whole tibial epiphysis while preserving intrinsic osteochondral interactions. We exploit the predictable onset of cartilage degeneration in male STR/Ort mice, which exhibit pathological features consistent with those observed in age‐related OA in humans, and compare these joints with those of the parental CBA strain, which undergoes healthy aging [48, 49, 50] (Figure 1A). By combining multiscale strain quantification with comprehensive microarchitectural and computational analyses of these tibial epiphyses, we define, for the first time, mechano‐architectural features that preserve coordinated load dissipation during healthy aging and regional mismatches that are associated with osteoarthritic degeneration.

## 2 Results

### 2.1 Elevated Load‐Induced Strains Arise Prior to OA Emergence in the STR/Ort Tibial Epiphysis

To determine how load is distributed across tibial epiphyses with differing OA predisposition, we established a high‐resolution in situ strain mapping framework to quantify full‐field deformation in intact murine knee joints under physiological compression. This involved the implementation of a quasi‐static, displacement‐controlled loading protocol that recapitulates physiological knee joint loading, applied using a beamline‐compatible mechanical testing rig adapted from our previously described in vivo loading system [51] for phase‐contrast sCT imaging. Using this novel configuration, hindlimbs were initially stabilized under a 2 N baseline load in a flexed position [52] (Figure 1B). Owing to time‐dependent stress relaxation, consistent with the viscoelastic and poroelastic behavior of joint tissues, sCT image acquisition was performed 15 min after load application once force stabilization had been achieved. The greatest stress relaxation response was observed after the initial 2 N baseline load (Figure S1A–D). Subsequent sCT datasets were acquired at displacement increments of + 20, + 70, and + 170 µm relative to the 2 N baseline‐loaded state, with each displacement increment followed by the same relaxation period. As these displacement‐controlled steps were applied to preloaded, stress relaxed joints, each subsequent displacement step generated a smaller stress relaxation response than that observed following the initial 2 N baseline load. Peak forces ranged from 1.0–3.5 N with corresponding relaxed/imaged forces of 0.5–1.5 N (52%–63% reduction). Nevertheless, relaxed/imaged forces generally increased across successive displacement increments, consistent with our prior in situ loading studies [26]. Inspection of sCT images at baseline load confirmed preservation of epiphyseal microarchitecture in 10‐ and 40‐week‐old CBA mice and the development of progressive OA pathology in STR/Ort knee joints, characterized by SCP sclerosis, osteophytosis and joint space narrowing in 40‐week‐old animals (Figure S2A). Histological examination using the Osteoarthritis Research Society International scoring (OARSI) system (Figure S2B,C) confirmed the presence of intact tibial articular cartilage in 10‐week‐old STR/Ort and all CBA mice (Figure S2D), whereas marked medial (*p* = 0.03) and lateral (*p* = 0.01) cartilage lesions were evident in the tibial plateau of 40‐week‐old STR/Ort mice (Figure S2E). To quantify how these genotype‐ and age‐associated epiphyseal differences influenced load‐induced deformation, epiphyseal volumes were segmented from high‐resolution sCT images to generate FE tetrahedral meshes (Figure 1C–E). Nodal displacement fields were then computed using a global DVC algorithm that correlated grayscale intensity variations between baseline and loaded images. This workflow achieved a displacement accuracy of 18 nm (loading direction, z‐axis) and displacement precision of 78 nm, and mean strain accuracy and precision of 53 and 221 µstrain, respectively (see Supplementary Information).

**FIGURE 2:**
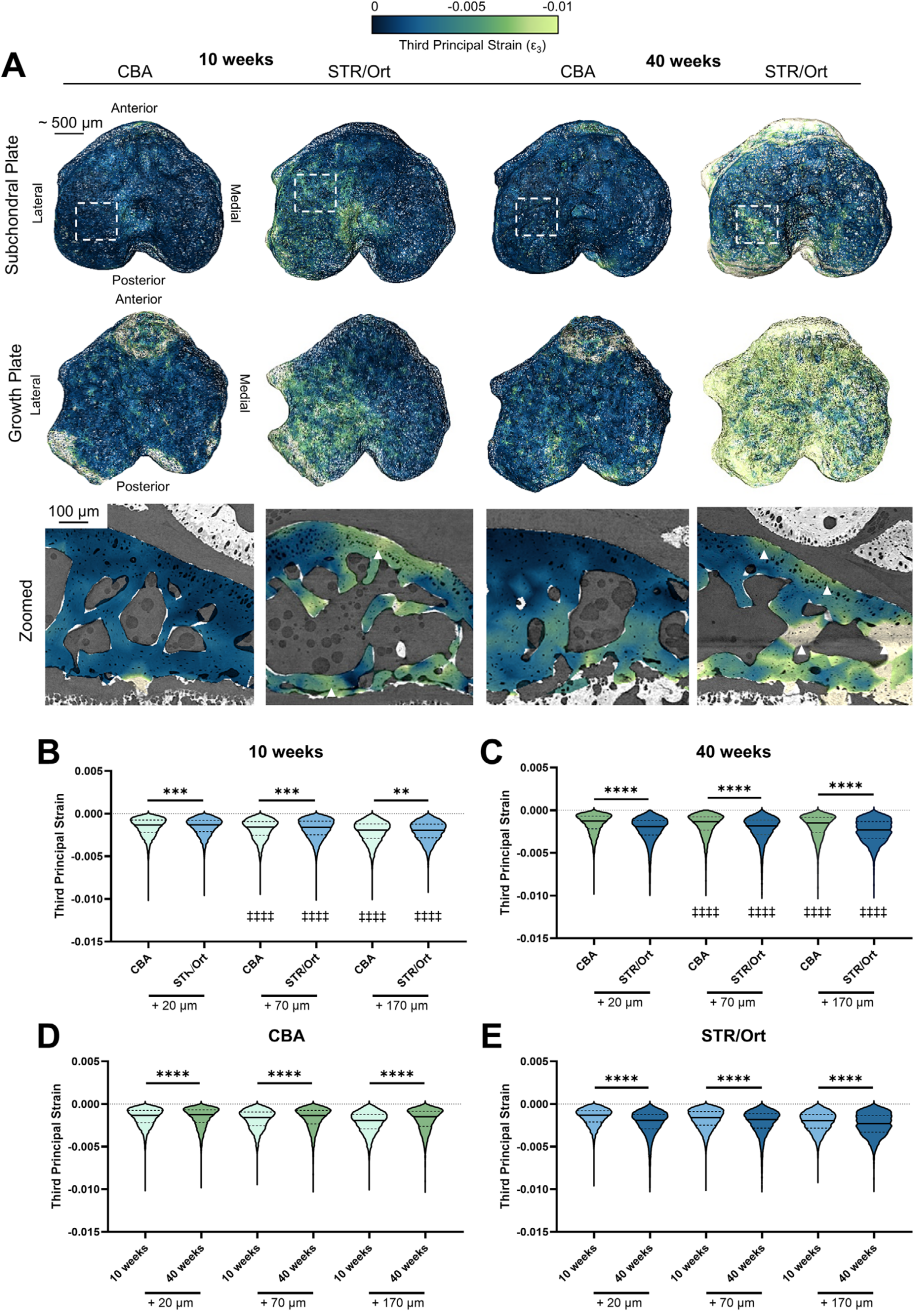
Emergence of OA pathology in STR/Ort mice is associated with the regionalized concentration of compressive strains. DVC‐computed strains are superimposed on FE tetrahedral meshes of 10‐week‐old (left) and 40‐week‐old (right) CBA and STR/Ort tibial epiphyses, viewed from the SCP (A, top row) and growth plate (A, bottom row), showing localization of high (cream) and low (dark blue) compressive strains (scale bar = 500 µm). In CBA mice, high magnitude compressive strains are confined to growth plate regions at both ages. In STR/Ort mice, relatively higher magnitude compressive strains are evident across the SCP and growth plate regions at 10 weeks of age and further increase by 40 weeks of age in line with OA progression. Zoomed sagittal sCT images (white hatched box) overlaid with compressive strain maps (A, bottom) highlight high strain concentration within the distal epiphysis in CBA mice and within the SCP and growth plate regions in STR/Ort mice (white arrowheads) (scale bar = 100 µm). Compressive strain distributions from DVC FE‐mesh nodes in response to displacement‐induced loading in 10‐week‐old (B) and 40‐week‐old (C) animals are presented as violin plots. The effect of age on compressive strain distribution is shown for CBA (D) and STR/Ort epiphyses (E) following incremental displacement. Individual violins represent pooled DVC‐derived strain distributions from N = 4 mice per age and genotype; the solid line indicates the median, and dashed lines indicate the 25^th^ and 75^th^ percentiles. Statistical differences in strain distributions between CBA and STR/Ort epiphyses were assessed using the Kolmogorov–Smirnov test (^**^ *p*<0.01, ^***^ *p*<0.001, and ^****^ *p*<0.0001). Statistical significance between displacement‐induced load steps relative to the + 20 µm displacement step was assessed using one‐way ANOVA with Šídák's post hoc test (‡‡‡‡*p*<0.0001).

Analysis of principal strains (compression, third principal; tension, first principal) revealed near‐uniform low compression across the SCP of 10‐week‐old CBA epiphyses (Figure 2A, left, top), with elevated strains confined to regions proximal to the growth plate (Figure 2A, left, bottom). In contrast, age‐matched STR/Ort epiphyses exhibited modestly elevated and laterally concentrated compressive strains in SCP and growth plate regions (arrowheads). Compression patterns observed in 10‐week‐old CBA epiphyses remained comparable by 40 weeks of age (Figure 2A, middle), whereas STR/Ort epiphyses developed pronounced focal strain accumulation within the SCP and heightened deformation at the growth plate, reflecting increased epiphyseal deformability (Figure 2A, right, bottom).

Quantitative analysis confirmed these visual observations. At + 20 µm displacement, STR/Ort epiphyses displayed significantly different compressive strain distributions from CBA controls at 10 weeks of age (Kolmogorov–Smirnov D = 0.025, *p* = 0.0004; Figure 2B), with differences persisting at higher displacements (+ 70 and + 170 µm; D = 0.026, *p* = 0.0002 and D = 0.023, *p* = 0.002, respectively). By 40 weeks, differences between CBA and STR/Ort compressive strain distributions were more pronounced across all displacements (D = 0.18–0.24, all *p*<0.0001; Figure 2C). Median compressive strains and interquartile ranges (IQRs) were consistently elevated in STR/Ort epiphyses at both ages (Figure 2B,C and Table S1), whereas CBA mice exhibited only modest age‐related shifts in strain distribution (D = 0.04–0.15, *p*<0.0001; Figure 2D). Increasing displacement amplified compressive strain magnitudes in both genotypes, with higher values in young compared with aged CBA epiphyses. Aging in STR/Ort mice produced smaller yet significant shifts in compressive strain distribution (D = 0.1–0.21, *p*<0.0001; Figure 2E), with minimal changes in median strain and variability (Table S1). Thus, identical applied displacement increments produced a greater magnitude and range of compressive strains in STR/Ort epiphyses.

Tensile strain behavior mirrored these genotype‐dependent trends (Figure S3A–C and Table S2). CBA epiphyses displayed pronounced age‐related redistribution of tensile strain (D = 0.32–0.42, *p*<0.0001; Figure S3D), with higher median values at 10 weeks of age (Table S2). In contrast, STR/Ort epiphyses exhibited larger load‐induced shifts in tensile strain distribution (D = 0.04–0.26, Figure S3E), without age‐related changes in median strain or IQR (Tables S1 and S2). Across genotypes and ages, increasing displacement significantly increased both compressive and tensile strain magnitudes relative to the + 20 µm displacement step (all *p*<0.0001; Figure 2B,C and Figure S3B,C, respectively). Collectively, these findings indicate that OA‐prone STR/Ort epiphyses exhibit amplified and spatially confined deformation under compressive loading prior to overt cartilage degeneration, whereas strain patterns in CBA joints reflect a regulated distribution characteristic of healthy aging.

### 2.2 Joint Loading Creates Laterally Biased Deformation in the STR/Ort SCP

**FIGURE 3:**
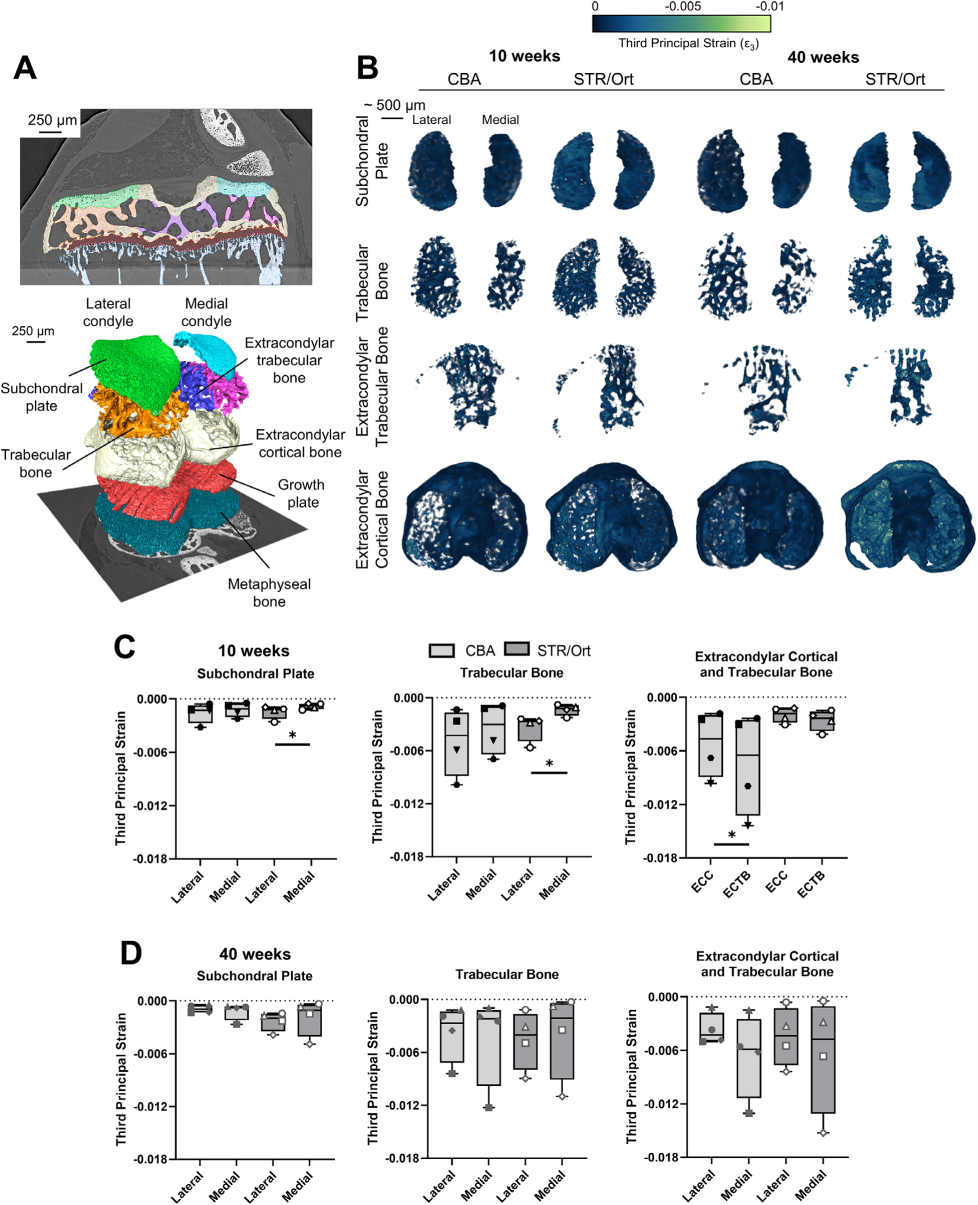
Regionalized strain quantification reveals imbalanced distribution of compressive strain across STR/Ort epiphyseal condyles. Segmentation of anatomical compartments within the lateral and medial condyles of the tibial epiphysis, including the SCP, trabecular bone, ECTB, and ECC (A), is shown in 2D coronal sCT images (top) and 3D renders (bottom; scale bar = 250 µm). Region‐specific DVC‐derived compressive strain fields are displayed as 3D renders of the SCP, trabecular bone, ECTB, and ECC from 10‐ and 40‐week‐old CBA and STR/Ort mice (B; scale bar = 500 µm). Quantification of average compressive strain in the lateral and medial condylar compartments of the tibial epiphyses of 10‐week‐old (C) and 40‐week‐old (D) CBA and STR/Ort mice show that laterally dominant load‐induced condylar strains arise in 10‐week‐old STR/Ort mice, whereas lateromedial balancing of strain is evident in CBA mice at both ages. Data are presented as box and whisker plots, where boxes represent the IQR, the central line denotes the median, and whiskers correspond to the minimum and maximum values. Symbols represent individual animals (N = 4 per age and genotype). Statistical significance between regions was assessed using two‐way ANOVA with Šídák's post hoc test (^*^ *p*<0.05, ^**^ *p*<0.01).

Previous work established that our loading configuration preferentially transmits loads through the lateral joint compartment [51]. To determine whether early strain amplification in STR/Ort epiphyses exhibits regional bias under these loading conditions, DVC datasets were subdivided into lateral and medial anatomical compartments (Figure 3A). In CBA epiphyses, both compressive and tensile strains were low and symmetrically distributed across the SCP and trabecular regions at both ages (Figure 3B and Figure S4A). In contrast, STR/Ort epiphyses displayed a pronounced lateral bias in both strain modes within the SCP, with heightened strains extending into the extracondylar cortical bone (ECC) at 10 weeks of age; this pattern became more evident by 40 weeks of age.

Quantitative analysis confirmed balanced lateral‐medial compressive (Figure 3C) and tensile (Figure S4B) strains in the SCP of 10‐week‐old CBA mice, whereas STR/Ort epiphyses experienced significantly greater compression and tension in the lateral SCP (both *p* = 0.04). This lateral dominance was also observed in the condylar trabecular bone of STR/Ort mice for both strain modes (compression, *p* = 0.01; tension, *p* = 0.02). In the extracondylar regions, compressive strains were higher in the trabecular compartment (ECTB) than in the cortical bone compartment (ECC; *p* = 0.05) in CBA mice, whereas tensile strains predominated in the ECTB of STR/Ort epiphyses (*p* = 0.008). By 40 weeks of age, compressive and tensile strains were lateromedially balanced across compartments in both genotypes (Figure 3D and Figure S4C). These data demonstrate that lateral strain dominance in STR/Ort epiphyses is compartmentally confined and constitutes an early imbalance in load transmission, whereas symmetric strain transfer is preserved during healthy joint aging.

Regional FE modeling (Figure S5A) yielded compartment‐specific material property estimates that showed >95% agreement with DVC‐derived strains (Figure S5B,C), with only 1.4%–2.3% deviation at high‐strain nodes (Figure S5D,E, respectively). In 10‐week‐old CBA epiphyses, lateral and medial SCP and trabecular bone moduli were comparable (Table 1), whereas the ECTB was significantly less stiff than the ECC (*p* = 0.009). STR/Ort epiphyses of the same age also exhibited lateromedial symmetry in SCP and trabecular bone moduli, with reduced ECTB stiffness relative to ECC (*p* = 0.02). Notably, SCP stiffness was significantly lower in STR/Ort than CBA mice at 10 weeks of age in both the lateral (*p* = 0.002) and medial (*p* = 0.0002) condyles, consistent with elevated compressive strain in STR/Ort joints. This was accompanied by greater compliance in the ECTB than ECC (*p* = 0.05). Together, these stiffness differences may partly explain the elevated deformability observed in STR/Ort epiphyses; however, given that these differences were not restricted to the lateral compartment, stiffness changes alone do not fully explain the laterally biased strain dominance.

**TABLE 1:** FE predictions of Young's modulus and Poisson's ratio for 10‐ and 40‐week‐old CBA and STR/Ort mice across anatomical compartments of the tibial epiphysis. Data are presented as mean ± SD for N = 3 mice per age and genotype. Statistical significance was assessed using two‐way ANOVA with Šídák's post hoc test. † denotes differences between 10‐ and 40‐week‐old animals, ^*^ denotes differences between CBA and STR/Ort mice, and § denotes differences between epiphyseal condyles/compartments; single, double, and triple symbols correspond to *p*<0.05, *p*<0.01, and *p*<0.001, respectively.

			Lateral Subchondral Plate	Medial Subchondral Plate	Lateral Trabecular Bone	Medial Trabecular Bone	Extracondylar Cortical Bone	Extracondylar Trabecular Bone
Predicted Modulus (GPa)	10 weeks	CBA	3.32 ± 0.49	3.8 ± 0.2	7.84 ± 0.96	8.78 ± 0.81	9.47 ± 1.27	5.38 ± 0.55 §§
		STR/Ort	1.10 ± 0.67 **	0.86 ± 0.58 ***	7.43 ± 2.0	7.86 ± 3.33	10.77 ± 0.59	7.51 ± 1.20 §, *
	40 weeks	CBA	3.51 ± 0.8	3.96 ± 0.29	7.49 ± 0.58	5.78 ± 2.0 †	10.58 ± 1.26	5.36 ± 1.38 §§
		STR/Ort	4.13 ± 0.68 †††	3.48 ± 0.25 ††	1.24 ± 0.89 †, ***	2.18 ± 0.99 †, *	7.83 ± 2.11	2.89 ± 0.92 ††, §§
Predicted Poisson's Ratio (A.U.)	10 weeks	CBA	0.24 ± 0.01	0.22 ± 0.01	0.26 ± 0.03	0.27 ± 0.06	0.22 ± 0.04	0.29 ± 0.02 §
		STR/Ort	0.2 ± 0.02	0.25 ± 0.02	0.24 ± 0.03	0.28 ± 0.03	0.21 ± 0.02	0.29 ± 0.01 §§
	40 weeks	CBA	0.23 ± 0.01	0.21 ± 0.02	0.29 ± 0.03	0.29 ± 0.04	0.24 ± 0.03	0.29 ± 0.01
		STR/Ort	0.22 ± 0.02	0.22 ± 0.01	0.27 ± 0.03	0.24 ± 0.02	0.22 ± 0.01	0.3 ± 0.04 §

Aging in CBA epiphyses was characterized by preservation of SCP moduli in both condyles, with reductions evident only in the medial trabecular compartment (*p* = 0.03). Conversely, aging in STR/Ort epiphyses was associated with increased stiffness of both the lateral and medial SCP (*p* = 0.0004 and *p* = 0.001, respectively), alongside reductions in trabecular bone modulus in both condyles (lateral, *p* = 0.01 and medial, *p* = 0.02, respectively). ECTB stiffness remained lower than ECC stiffness in both CBA (*p* = 0.003) and STR/Ort epiphyses (*p* = 0.004). By 40 weeks of age, lateral and medial trabecular bone modulus was significantly lower in STR/Ort than CBA mice (*p* = 0.0005 and *p* = 0.01, respectively).

Predicted Poisson's ratio remained largely conserved across compartments, with only modest reductions in the ECC relative to the ECTB in 10‐week‐old CBA and STR/Ort epiphyses (*p* = 0.01 and *p* = 0.007, respectively) and in 40‐week‐old STR/Ort epiphyses (*p* = 0.01). Taken together, these findings identify STR/Ort epiphyses as mechanically imbalanced structures in which compartment‐specific stiffness alterations modulate load‐bearing behavior. This regionalized mechanical asymmetry aligns with the strain amplification resolved in response to compressive loading and implicates structural organization, rather than global material deficiency, as a determinant of early mechanical vulnerability.

### 2.3 Epiphyseal Microarchitecture Predicts Susceptibility to Load‐Induced SCP Deformation Prior to OA

**FIGURE 4:**
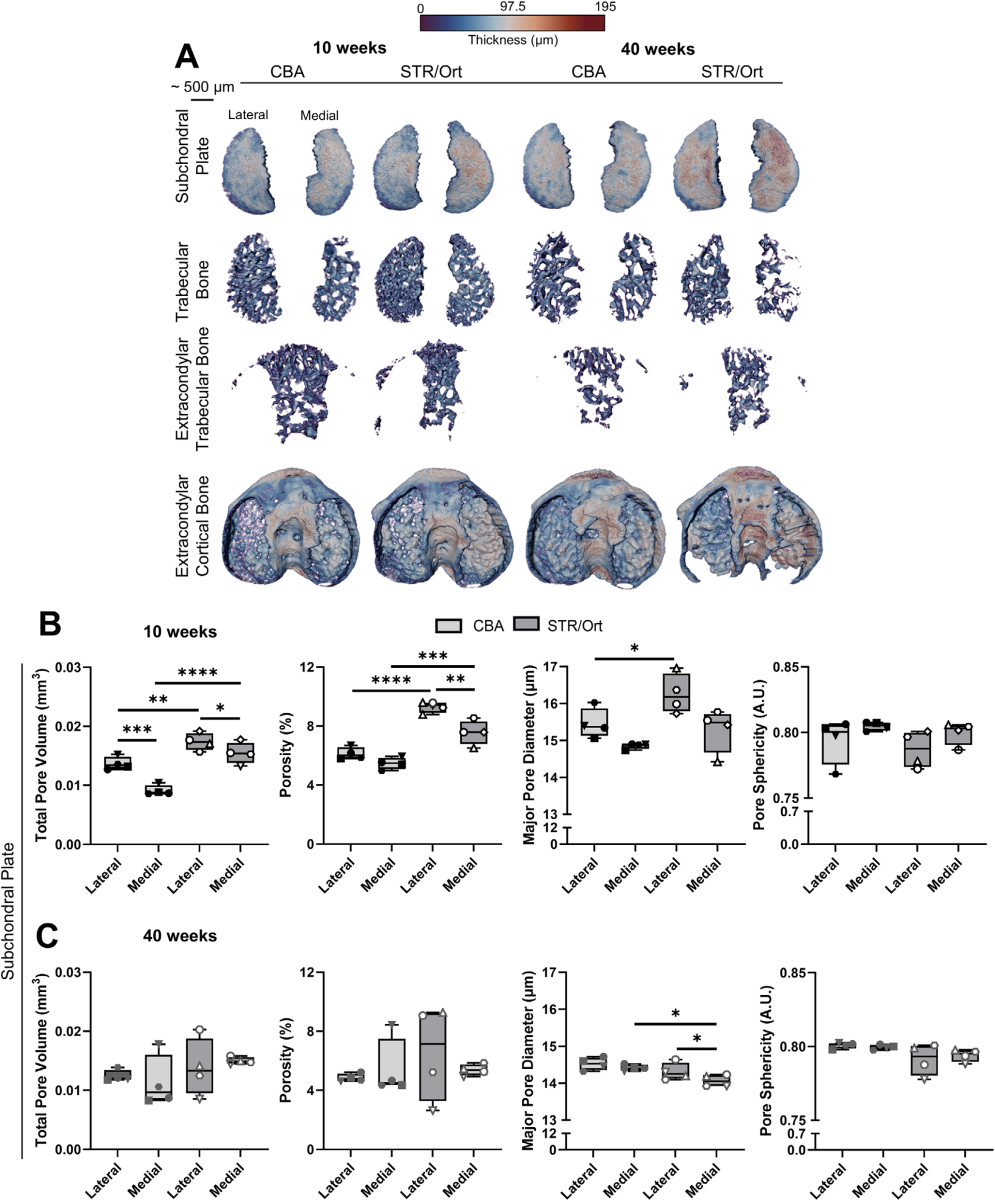
STR/Ort tibiae exhibit microstructural alterations in the epiphyseal SCP that precede age‐related OA. Volumetric thickness maps of the SCP, condylar trabecular bone, ECTB, and ECC are shown for 10‐ and 40‐week‐old CBA and STR/Ort epiphyses (A, scale bar = 500 µm). Quantification of total pore volume, porosity, major pore diameter, and pore sphericity reveals greater pore volume and porosity in STR/Ort epiphyses at 10 weeks of age (B), which is no longer evident by 40 weeks of age (C). Data are presented as box and whisker plots, where boxes represent the IQR, the central line denotes the median, and whiskers correspond to the minimum and maximum values. Symbols represent individual animals (N = 4 per age and genotype). Statistical significance between regions was assessed using two‐way ANOVA with Šídák's post hoc test (^*^ *p*<0.05, ^**^ *p*<0.01, ^***^ *p*<0.001, ^****^ *p*<0.0001).

To determine whether regionalized strain asymmetry observed in STR/Ort epiphyses reflects an underlying architectural incongruity, we performed comprehensive microstructural analyses of CBA and STR/Ort epiphyses (Figure 4A and Table S3). At 10 weeks of age, STR/Ort mice exhibited significant medial SCP enlargement, with increased thickness and volume (Table S3, *p* = 0.0004 and *p* = 0.005, respectively), while the lateral SCP remained comparable to CBA mice. In contrast, asymmetry in CBA mice was limited to the medial SCP, which had greater volume than the lateral SCP (*p*<0.0001). Within the medial condyle of STR/Ort mice, the cancellous bone compartment contained thicker trabeculae but occupied reduced overall volume compared with the lateral condyle (*p* = 0.002 and *p* = 0.003, respectively) and remained larger than that of CBA mice (volume; *p* = 0.01). Epiphyseal trabecular thickness was comparable between CBA condyles, despite lower medial trabecular bone volume (*p* = 0.0005). The ECC was thicker and lower in volume than the ECTB in both 10‐week‐old CBA and STR/Ort epiphyses (all *p*<0.0001), with STR/Ort ECCs also thicker than CBA ECCs (*p* = 0.009).

In 40‐week‐old STR/Ort mice, medial SCP thickening persisted and was characterized by greater thickness (*p* = 0.02) and volume (*p* = 0.05) relative to the lateral SCP, with age‐related thickening evident only in the medial SCP (*p* = 0.04). This was accompanied by asymmetric enlargement of the lateral SCP (volume; *p* = 0.004). In CBA mice, the medial SCP also became thicker and larger with age (*p* = 0.04 and *p* = 0.002, respectively), although the SCP volume remained greater in 40‐week‐old STR/Ort epiphyses than in CBA epiphyses in both condyles. Aging reduced lateral condylar trabecular bone volume in both CBA (*p* = 0.003) and STR/Ort mice (*p* = 0.007), while CBA mice retained greater lateral than medial trabecular bone volume (*p* = 0.04). Marked extracondylar corticalization occurred with age in both strains, reflected by increased ECC thickness and volume in CBA mice (*p*<0.0001 and *p* = 0.002, respectively) and STR/Ort mice (*p* = 0.01 and *p* = 0.02, respectively), without corresponding age‐related changes in ECTB. Collectively, these adaptations indicate that age‐related SCP thickening and extracondylar corticalization do not explain the heightened early deformability observed in STR/Ort epiphyses.

Closer interrogation of SCP microarchitecture revealed that total pore volume and percentage porosity were substantially greater in 10‐week‐old STR/Ort than CBA epiphyses across both condyles (total pore volume: lateral, *p* = 0.005 and medial, *p*<0.0001; percentage porosity: lateral, *p*<0.0001 and medial, *p* = 0.0003, respectively) (Figure 4B). While lateral pore volume exceeded medial SCP pore volume in both genotypes (CBA, *p* = 0.0003 and STR/Ort, *p* = 0.02), percentage porosity was selectively elevated in the lateral SCP of STR/Ort epiphyses (*p* = 0.007). These lateral SCP pores additionally had greater diameters in STR/Ort than CBA mice (*p* = 0.05), while pore sphericity remained comparable between genotypes at this age. By 40 weeks of age, total pore volume, percentage porosity, and pore sphericity were similar between the CBA and STR/Ort SCPs (Figure 4C), although medial SCP pore diameter was lower than lateral SCP pore diameter in STR/Ort mice (*p* = 0.04) and greater in CBA mice (*p* = 0.03). These findings reveal regionalized asymmetry in SCP architecture, characterized by biased porosity and altered pore geometry in STR/Ort epiphyses, which may contribute to focal strain amplification under compressive loading. Notably, these microstructural incongruities precede overt cartilage degeneration and are not attributable to uniform increases in epiphyseal bone mass, implicating regional subchondral architecture as a determinant of early mechanical vulnerability in OA‐prone joints.

**FIGURE 5:**
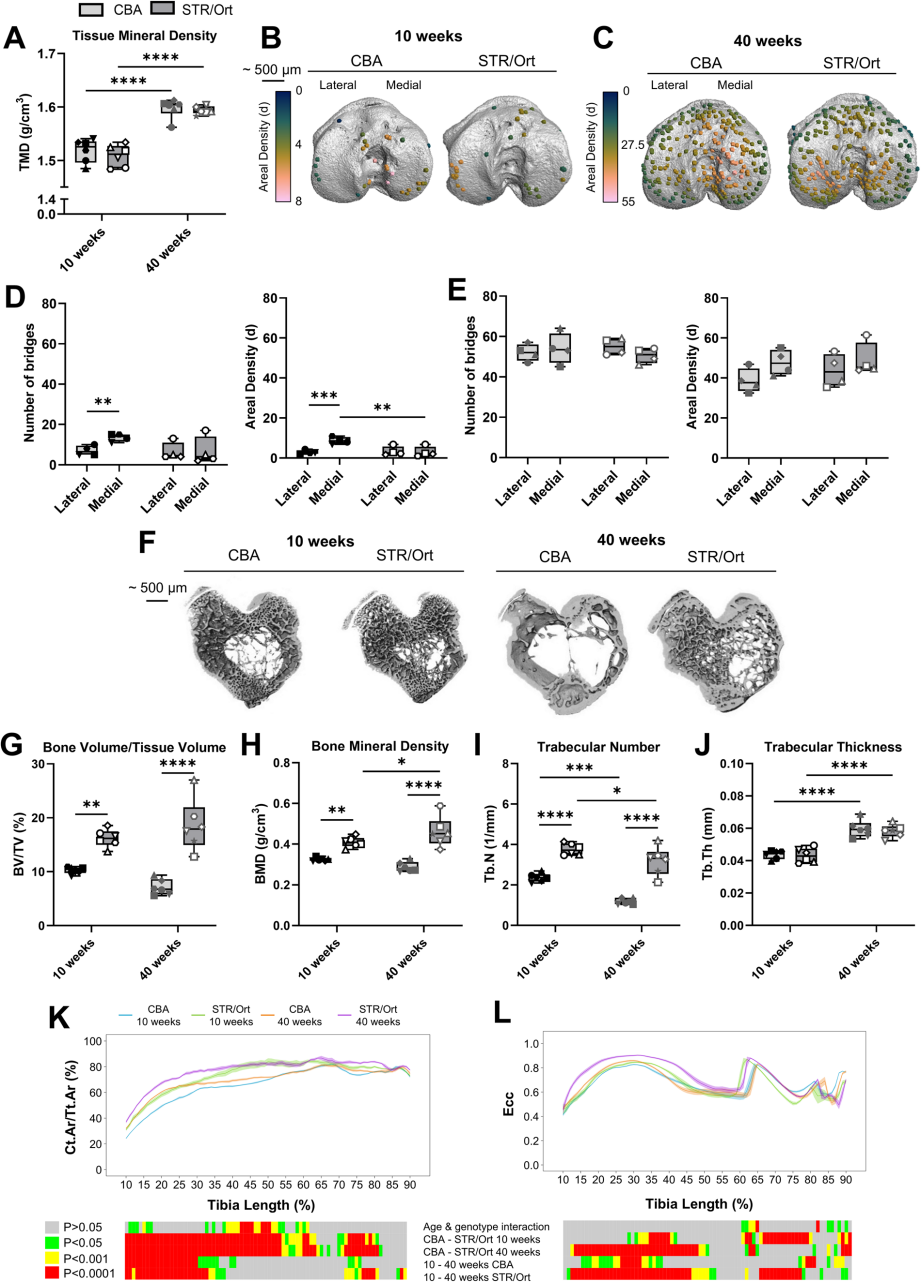
Enhanced metaphyseal, but not epiphyseal, bone mass is accompanied by proximal curvature in STR/Ort epiphyses. µCT‐based tibial morphometric analyses reveal comparable epiphyseal TMD between CBA and STR/Ort mice of the same age, with TMD increasing with age in both genotypes (A). Examination of the location and areal densities of growth plate bridges in 10‐ and 40‐week‐old CBA (B) and STR/Ort mice (C; scale bar = 500 µm) reveals greater medial enrichment of bridge number and areal density in CBA mice at 10 weeks of age, whereas STR/Ort mice display lateromedially balanced bridge distribution and reduced medial bridging relative to CBA mice (D). With aging, bridge number and areal density increase across condyles in both genotypes, resulting in comparable distributions by 40 weeks of age (E). Representative 3D renderings of reconstructed µCT images of the tibial metaphyseal trabecular bone in 10‐ and 40‐week‐old CBA and STR/Ort mice are shown (F; scale bar = 500 µm). Morphometric analyses of metaphyseal trabecular BV/TV (G), BMD (H), Tb.Th (I), and Tb.N (J) indicate that the high trabecular bone mass phenotype in STR/Ort mice is maintained with age. Data are presented as box and whisker plot for A, D, E, G‐J, where boxes represent the IQR, the central line denotes the median, and whiskers correspond to the minimum and maximum values; symbols represent individual animals (N = 4 per age and genotype for D and E and N = 6 per age and genotype for A, (G–J). Statistical significance between regions and ages was assessed using two‐way ANOVA with Šídák's post hoc test (^*^ *p*<0.05, ^**^ *p*<0.01, ^***^ *p*<0.001 and ^****^ *p*<0.0001). Tibial Ct.Ar/Tt.Ar (K) and eccentricity (Ecc, L), evaluated along 10%–90% of tibial length, are presented as line graphs showing mean ± SEM. Heatmaps beneath each plot show statistical significance at spatially matched locations along the tibial length with age (10 vs. 40 weeks) and between genotypes (CBA vs. STR/Ort; N = 6 per age and genotype). Statistical significance was assessed using two‐way ANOVA with Tukey's post hoc test (K and L; gray, *p*>0.05; green, *p*<0.05; yellow, *p*<0.001; and red, *p*<0.0001).

To determine whether tibial morphology contributes to the epiphyseal strain asymmetry observed in STR/Ort joints, whole‐tibia morphometry was assessed by µCT. Epiphyseal tissue mineral density (TMD, Figure 5A) was comparable between genotypes but increased with age in both groups (*p*<0.0001) and was not influenced by differences in epiphyseal volume (Figure S6A). At 10 weeks of age, CBA mice showed medial enrichment of growth plate bridges, with greater bridge number (*p* = 0.006) and areal density (*p* = 0.001) than the lateral side (Figure 5B,D). In contrast, while bridge number did not differ between condyles in STR/Ort mice, medial bridge areal density was lower relative to CBA mice (*p* = 0.003). With aging, bridge number and density increased across condyles in both genotypes (*p*<0.0001), resulting in comparable distributions by 40 weeks of age (Figure 5C,E).

STR/Ort mice exhibited a high metaphyseal trabecular bone mass phenotype (Figure 5F), with greater bone volume/tissue volume (BV/TV; *p* = 0.002, Figure 5G), bone mineral density (BMD; *p* = 0.004, Figure 5H) and trabecular number (Tb.N; *p*<0.0001, Figure 5I) at 10 weeks of age compared with CBA mice. Healthy CBA aging was characterized by increased metaphyseal trabecular thickness (Tb.Th; *p*<0.0001, Figure 5J), and reductions in Tb.N (*p* = 0.0001), whereas STR/Ort mice displayed modest increases in BMD and Tb.Th (*p* = 0.05 and *p*<0.0001, respectively), with reductions in Tb.N (*p* = 0.04). Additional trabecular microstructural parameters, including trabecular spacing (Tb.Sp), trabecular pattern factor (Tb.Pf), anisotropy, and connectivity density (Conn.D), also varied by genotype and age (Figure S6B–F). Tibial length was comparable between genotypes at both 10 and 40 weeks of age (Figure S6G,H), but increased with age in both CBA and STR/Ort mice (both *p*>0.0001). STR/Ort tibial diaphyses had greater cortical area (Ct.Ar/Tt.Ar) and proximal eccentricity than CBA mice at both ages (Figure 5K,L). Several other cortical shape metrics, however, varied predominantly with age (Figure S6I–L). Collectively, these morphometric analyses suggest that high tibial bone mass and altered tibial diaphyseal geometry in STR/Ort mice may influence the mechanical response of the knee joint to compressive loading. Together with regional changes in SCP architecture, these features may reduce epiphyseal mechanical competence and increase susceptibility to load‐induced failure in OA‐prone joints.

## 3 Discussion

It is widely recognized that joint microstructure and mechanical behavior are interdependent, yet the mechanisms that underpin physiological load transfer across the healthy knee joint to preserve mechanical competence have remained unresolved. This has consequently limited insight into the relative contributions of age‐related tissue deterioration and alterations in load transfer across this osteochondral system in OA. By combining in situ mechanical loading with phase‐contrast sCT and DVC, we resolve the full‐field strain landscape of the intact murine tibial epiphysis in 3D under physiologically relevant loading conditions for the first time. Our findings reveal a clear mechanical dichotomy between healthy aging and OA‐prone joints. In the epiphyses of healthy CBA mice, architectural integration across the SCP, trabecular bone and epiphyseal cortices permits uniform strain distribution, diverting peak compressive strains away from the articulating surface. In contrast, STR/Ort epiphyses exhibit focal and laterally biased strain amplification within the SCP at 10 weeks of age, coincident with regionally biased porosity, asymmetric thickening, and reduced SCP stiffness before the onset of cartilage degeneration. These features are consistent with, but not directly equivalent to, subchondral structural heterogeneity and altered mechanical behavior reported in human OA [53, 54]. Importantly, whole‐tibia morphometry revealed additional metaphyseal and diaphyseal divergence in STR/Ort mice, supporting the concept that early mechanical vulnerability in this model reflects multiscale architectural incongruity that disrupts coordinated load transfer across the epiphysis.

Previous non‐invasive in vivo loading models have been instrumental in defining skeletal adaptation to mechanical challenges. In the tibial loading model [51], dynamic compressive loads of 12 N induce periosteal expansion, cortical thickening, and changes to metaphyseal trabecular architecture, whereas single or repeated 9 N loads are sufficient to induce only focal cartilage injury and OA‐like changes laterally [52]. Although invaluable for studying mechanoadaptation, these approaches do not resolve the internal 3D strain fields that arise within intact osteochondral tissues during loading, nor do they permit separation of architectural determinants from functional mechanical behavior during OA progression. More recent advances in sCT have permitted exploration of the real‐time microstructural response of bovine cartilage and meniscus plugs to axial loads [55], demonstrating that tissue deformation is linked to the reorganization of chondrocyte orientation and collagen fibers along the loading direction. While mechanistically informative, investigations on this scale do not reveal how local material properties and microarchitecture integrate to govern organ‐level load transfer. Our earlier sCT‐based in situ feasibility studies using DVC [26] addressed this limitation and suggested that osteochondral strain propagation in STR/Ort knee joints may be influenced by distinct chondrocyte lacunar organization, a feature observed in human OA and associated with reductions in local tissue elasticity [56, 57].

Building on these advances, the present study adapts the non‐surgical knee loading model [52] for displacement‐controlled in situ loading and sCT of larger numbers of intact hindlimbs, enabling full‐field epiphyseal strain mapping by DVC. This was achieved using anatomically matched loading cups to preserve physiological femur‐tibia alignment and habitual load paths reflective of native joint motion. Given that loading was displacement‐controlled, the applied displacement steps cannot be translated into a single defined joint stress, since stress distribution depends on joint geometry, contact area, tissue composition and regional structural heterogeneity. Instead, the applied displacement increments generated measured peak loads of approximately 1–3.5 N and relaxed/imaged loads of approximately 0.5–1.5 N following stress relaxation. These values are consistent with our prior in situ loading experiments [26] and remain below ex vivo tibial loads reported to generate strains comparable to those measured during physiological limb use [51], while enabling full‐field strain measurements by DVC. In addition to the load magnitudes reported above, the loading protocol itself merits consideration, as a quasi‐static approach was required to mitigate viscoelastic drift and maintain joint stability during sCT acquisition. The initial 2 N baseline load generated a higher peak force than subsequent displacement‐controlled loading steps. This likely reflects joint preconditioning by the baseline load, which was necessary to stabilize the knee and ankle of the hindlimb in deep flexion between the load cups [51, 52] and establish a reproducible baseline knee configuration. This aspect of our loading approach represents a limitation of the study, as the relatively high preload may influence the subsequent mechanical response of the joint, and, in turn, the reported DVC‐derived strain fields and FE‐derived elastic moduli. Nevertheless, the relaxed/imaged loads generally increased with stepwise displacement across all samples, indicating progressive deformation of the knee joint and providing suitable loading states for DVC‐based displacement and strain quantification. This approach thereby enabled regional nanoscale strain heterogeneity to be resolved across the entire tibial epiphysis under physiologically relevant loading conditions, preserving osteochondral continuity and enabling interrogation of the architectural determinants underlying organ‐level load transfer.

STR/Ort mice are an extensively characterized model of age‐related OA, in which disease reliably emerges in the medial tibial plateau of males [48] at 18–20 weeks of age [49, 50, 58, 59, 60], characterized by ensuing subchondral sclerosis and osteophytosis, features comparable to those observed in primary human OA [50]. Early defects in endochondral ossification and dysregulation of molecular pathways involving sclerostin, NF‐κB signaling, the SIBLING protein family, and matrix metalloproteinases have been implicated in OA onset in this model [29, 49, 58], with several of these drivers conserved in other rodent OA models [61, 62, 63, 64, 65, 66]. However, OA has historically been regarded as a mechanically driven disease [67], and accumulating evidence suggests that many of these molecular alterations are coupled to, and likely secondary to, an underlying mechanical insufficiency [65, 67]. In STR/Ort mice, joint instability, medial patella dislocation, and anterior cruciate ligament (ACL) weakening associated with raised MMP‐2 expression [58], are thought to accelerate OA through gait modification [68], while cyclic loading exacerbates medial cartilage loss and subchondral bone thickening [49, 69]. This periarticular contribution is also evident in human OA, where ACL degeneration is highly prevalent in knees with cartilage defects [70], and increasing OA grade is associated with reduced ACL yield and failure stress [71]. Additional studies in STR/Ort mice have reported elevated local and systemic inflammatory markers, including serum IL‐1β, IL‐12p70, MIP‐1β, and IL‐5, as well as alterations in oxidative stress‐associated mediators, including HMGB1, advanced glycation end products, and extracellular superoxide dismutase [72, 73, 74], several of which have also been implicated in human OA pathogenesis [75, 76, 77]. In contrast, CBA mice exhibit minimal spontaneous OA with aging despite susceptibility to focal cartilage lesions following traumatic in vivo loading [49]. Our findings place these models within a unified mechanical context, showing that STR/Ort epiphyses display early intrinsic load transfer imbalances that are absent in CBA mice and manifest as spatially confined high magnitude strains within mineralized epiphyseal compartments prior to overt cartilage pathology. This suggests that mechanical vulnerability is encoded in epiphyseal architecture and precedes molecular or histological disease manifestations, although inflammatory processes and periarticular tissue changes may also contribute to this mechanical phenotype. We therefore propose that mechano‐architectural divergence represents an upstream determinant of OA predisposition in this OA‐prone mouse model.

Building on our prior in situ strain mapping, which observed localized load induced deformation within the epiphyseal subchondral bone of CBA mice and calcified cartilage in STR/Ort joints [26], our DVC analyses reveal spatially heterogeneous strain distributions in these OA‐prone murine epiphyses beyond the osteochondral junction. At presumptive pre‐OA stages, STR/Ort joints exhibit spatially asymmetric condylar strain amplification within the SCP, whereas healthy CBA epiphyses dissipate physiological deformation uniformly through the SCP, trabecular network and adjacent cortices, transmitting peak strains toward the proximal growth plate and away from the articulating surface. Under identical loading conditions, elevated and laterally concentrated SCP strain is generated in STR/Ort epiphyses, consistent with established lateral‐posterior load transmission patterns in this model [52]. Together, these findings indicate that disrupted coordination of strain distribution, rather than global overloading, defines early mechanical vulnerability in this model and positions subchondral asymmetry as an early mechanical feature associated with OA susceptibility. While non‐mineralized tissues of the knee joint, including articular cartilage, ligaments and synovial tissues, likely contributed to the strain distribution across the tibial epiphysis, quantitative measurement of soft tissue strain was beyond the resolution and contrast capabilities of our imaging approach. To define how prevailing loads are partitioned between these components, future studies should incorporate soft tissue‐resolved sCT imaging with our in situ loading approach and apply DVC to generate experimental displacement fields capable of validating FE‐based multiphasic contact models.

Although OA has long been framed as a primary disorder of articular cartilage, converging evidence from human and animal studies indicates that subchondral bone undergoes some of the earliest detectable pathogenic alterations prior to cartilage loss [29, 69, 78, 79, 80, 81, 82, 83, 84]. This temporal relationship is supported by clinical magnetic resonance imaging (MRI) studies documenting increased bone turnover in asymptomatic patients with early OA despite intact cartilage [85]. At later disease stages, patients with late stage OA [86] and small animal models with surgically induced OA [87, 88] exhibit enhanced cartilage matrix turnover, reflected by elevated C‐terminal telopeptide of type II collagen (CTX‐II), without a corresponding rise in bone resorption, as indicated by comparable levels of C‐terminal telopeptide of type I collagen (CTX‐I). Similarly, STR/Ort mice exhibit greater BMD and elevated urinary CTX‐II and hydroxylysylpyridinoline levels, indicating increased cartilage matrix turnover prior to radiographic disease onset, while deoxypyridinoline levels remain comparable to CBA mice at all disease stages [89]. Mechanistically, such compartment‐specific remodeling may involve osteoblast dysfunction in response to aberrant mechanical stress. Single‐cell transcriptomic profiling during bone marrow lesion development, a key driver of joint pain and structural progression in OA [90], revealed proteostasis impairment in subchondral osteoblasts, resulting in type I collagen misfolding and accumulation, alongside WNT5A‐mediated promotion of chondrocyte hypertrophy and death [91]. Future studies combining spatially resolved strain mapping with local molecular, cellular and metabolic readouts will be required to link subchondral microarchitecture, mechanical function and biological activity during OA progression.

The subchondral microstructural alterations observed in the present study are consistent with findings from human imaging and µCT studies of OA. Clinical MRI and hybrid imaging approaches have demonstrated that subchondral bone remodeling is spatially linked to regional cartilage loss, bone marrow lesions, and altered joint loading in early disease [92, 93]. Ex vivo high‐resolution peripheral quantitative computed tomography (HR‐pQCT) and µCT studies further show that increased SCP and trabecular thickening, together with reduced trabecular separation in load‐bearing regions, correlate with cartilage degeneration and disease severity in human knee OA [35, 94, 95, 96]. Collectively, these findings support the relevance of the microstructural and mechanical alterations identified in the STR/Ort model, particularly in the context of load‐driven subchondral adaptation during early OA.

Importantly, these clinically observed microstructural features are not merely descriptive markers of disease but are accompanied by structural changes with direct mechanical consequences. Focal alterations in SCP thickness, porosity, and mineralization beneath regions of cartilage degeneration have been shown to disrupt the underlying trabecular network [97, 98, 99, 100, 101, 102] while loading‐induced ectopic nanocrystal deposition at osteochondral interfaces increases local stiffness and alters structural coupling between the deep cartilage zones and subchondral bone [103]. Such localized architectural alterations are predicted to generate steep stiffness gradients and heterogenous load paths across these tissue junctions [18], a concept supported by our sCT‐DVC analyses. In young STR/Ort joints, regionally confined strain accumulation within the SCP reflects spatial differences in porosity that modify local tissue compliance and load distribution, producing asymmetries that cannot be attributed to material heterogeneity alone.

Although the molecular mechanisms underpinning these observations remain to be fully established, dysregulated subchondral remodeling in STR/Ort mice provides a plausible explanation for the spatially heterogeneous architectures observed here. Impaired osteoclast differentiation, attributed to reduced sensitivity of bone marrow cells to M‐CSF and RANKL [104], together with increased osteoblast activity [105], has been reported in STR/Ort mice. In addition, alterations arising from an inherent endochondral defect, including elevated sclerostin expression in emerging osteophytes and regions of subchondral sclerosis [106], the presence of larger hypertrophic chondrocytes [26], and elevated MMP‐13 expression [29], may contribute to the architectural divergence described here, although this requires further investigation.

Lateral and medial condylar stiffness was comparable in CBA epiphyses, whereas SCP stiffness was relatively lower in STR/Ort mice and consistent with the elevated and broadened strain fields observed under identical loading conditions. The FE‐derived Young's modulus values estimated here (∼0.2–11 GPa) are lower than reported tissue‐level values for murine tibial bone, which typically range from approximately 6 to 30 GPa depending on anatomical region, experimental method, loading mode, and modeling assumptions [107, 108, 109, 110]. This difference is expected given the inverse FE framework used here, in which homogenous, linear elastic, isotropic material properties were optimized for each segmented mineralized epiphyseal compartment to reproduce the DVC‐derived strain fields under idealized boundary conditions. DVC could not reliably resolve strains in articular cartilage, meniscus, ligaments or other periarticular tissues due to insufficient phase contrast; therefore, these tissues were not explicitly incorporated into our FE models. Their influence on the strain patterns reported here, however, cannot be excluded, and future studies capable of resolving soft tissue mechanics will be instrumental in defining their roles. Accordingly, the predicted material properties should be interpreted as effective regional parameters reflecting the integrated mechanical response of mineralized tibial epiphyseal compartments within intact joints within a quasi‐static loading regime, rather than intrinsic tissue‐level constants. This compartment‐resolved approach is expected to yield lower apparent moduli than bulk measurements, which are typically acquired from highly mineralized cortical bone, while retaining sensitivity to region‐specific stiffness differences relevant to the strain amplification observed in STR/Ort epiphyses.

Previous studies suggest that regional reductions in elastic modulus may arise from increased vascularization linked to microdamage accumulation [17, 99, 111], a feature implicated in advancing OA [81]. While trabecular stiffness was comparable between condyles, the presence of a lateral strain bias within the trabecular compartment suggests that altered SCP properties disproportionately influence load transmission into the underlying trabecular network in STR/Ort epiphyses. This spatial dissociation supports the concept that compartment‐specific mechanical modulation arises from SCP remodeling, which is thought to occur alongside or prior to epiphyseal trabecular remodeling during early OA [112]. By contrast, the predicted Poisson's ratio (∼0.2–0.3) remained consistent with reported and commonly assumed values for mineralized bone [113, 114, 115, 116, 117], supporting the plausibility of the optimized parameter space. The preservation of Poisson's ratio across regions and ages further supports selective alterations in stiffness rather than a global change in deformability. Importantly, extracondylar thickening in STR/Ort epiphyses did not appear to confer early mechanical protection, indicating that regional structural compensation does not restore coordinated compliance across OA‐prone tibial epiphyses. These findings refine prior reports that thickened articular cartilage in STR/Ort mice, which is evident at all ages, attenuates contact stresses at the articulating surface [118] despite reduced intrinsic cartilage stiffness [119]. Together, this highlights that altered joint mechanics in this model arise from coordinated but imbalanced tissue‐level adaptations rather than isolated material deficits. Although our FE framework employed idealized boundary conditions, convergence between DVC‐derived strain data and optimized regional properties supports a dominant role for subchondral remodeling in shaping the load‐bearing behavior of the tibial epiphysis. Future incorporation of experimentally measured compartment‐specific material properties will further refine these predictions.

Altered growth plate bridging provides additional context for the observed strain patterns and aligns with previous descriptions of abnormal epiphyseal fusion in STR/Ort mice [29]. In CBA epiphyses, medially enriched bridging at 10 weeks of age suggests that fusion of the growth plate is regionally initiated. Conversely, young STR/Ort epiphyses exhibited fewer and more symmetrically distributed bridges, suggesting altered spatial coordination between endochondral maturation and the developing mechanical environment. By 40 weeks of age, bridge number and density converged in both genotypes, implying that bridge accumulation may follow temporally distinct developmental trajectories that are not exclusively governed by prevailing strain patterns. As load‐responsive structures that define structural continuity across the tibial growth plate [120, 121, 122], growth plate bridges likely influence the directionality of stress transfer from the epiphysis to the metaphysis. In this context, the model proposed by Xie et al. [123], in which the secondary ossification center buffers hypertrophic growth plate chondrocytes from excessive stress, provides a mechanical explanation through which bridge disruption may subtly perturb stress continuity prior to skeletal maturation. The early divergence in bridge patterning observed in STR/Ort joints therefore raises the possibility that asymmetric load‐transfer is not solely degenerative but partially reflects a developmentally patterned architectural bias. Rather than acting as a primary driver, altered bridging patterns may reinforce existing compartmental strain heterogeneity by modifying axial load transfer across the epiphyseal‐metaphyseal interface.

When considered alongside compartment‐specific stiffness alterations and SCP porosity, divergence in long bone morphology further contextualizes epiphyseal mechanics in STR/Ort mice. Early medial SCP and trabecular thickening, adaptations typically associated with enhanced structural competence, are accompanied by increased lateral SCP porosity, pore volume and geometric abnormalities within the metaphyseal and diaphyseal tibial compartments [105, 124]. Combined, these differences may facilitate the redirection of axial loads toward discrete epiphyseal regions thereby altering physiological load‐transfer pathways with advancing OA. Accordingly, the phenotype reported here in STR/Ort mice represents an emergent manifestation of altered multiscale architectural organization, whereby spatially distributed structural variation across the epiphysis, metaphysis and diaphysis may contribute to the reorganization of physiological load‐transfer pathways and exacerbate joint vulnerability with age.

By demonstrating that structural heterogeneity precedes cartilage degeneration and influences organ‐level load transfer, our findings identify architectural asymmetry and focal strain concentration as early mechanical features of joint dysfunction in male STR/Ort mice. These changes may be relevant to early biomechanical alterations reported prior to symptomatic OA, including gait asymmetries and altered joint loading patterns in humans [125]. Emerging MRI‐based DVC methodologies capable of resolving full‐field strain in human joints and animal models [22, 126] further support the feasibility and translational relevance of this approach. Recent studies have identified ZDHHC11 palmitoyl transferase [127], DDX5 [128], osteoclast‐derived microRNAs [129], and kindlin‐2 [130] as candidate therapeutic targets in OA. Alongside these candidates, the HSP70‐proteasome axis and WNT5A signaling, identified as downstream effectors of aberrant subchondral mechanical stress [91], are particularly relevant given their role in coupling subchondral osteoblast dysfunction to chondrocyte fate. In this context, the mechanobiological framework presented here provides a scalable means to interrogate how genetic, developmental, or pharmacological perturbations influence joint‐level mechanical performance. Integration of structural imaging, strain mapping and computational modeling may therefore accelerate the functional validation of candidate disease‐modifying strategies in mechanically vulnerable joints across preclinical models and human tissues.

## 4 Conclusion

This study distinguishes healthy and pathological joint aging by demonstrating that coordinated epiphyseal load transfer is preserved in resilient joints but disrupted in OA‐prone mouse knee joints. By resolving full‐field strain across intact murine tibial epiphyses, we identify early OA‐associated joint dysfunction not as an inevitable consequence of aging, but as a mechanically imbalanced state associated with multiscale architectural divergence. Our framework, combining in situ mechanical testing with sCT and DVC, provides a quantitative basis for identifying joint vulnerability prior to irreversible cartilage degeneration and could be extended to larger preclinical models to investigate conserved mechanisms of load‐induced failure.

## 5 Experimental Methods

### 5.1 Animals and Sample Preparation

All procedures were performed in accordance with the UK Animals (Scientific Procedures) Act of 1986, UK Home Office Regulations under License Number P253385BB2, and local institutional guidelines at the Royal Veterinary College, UK. Male STR/Ort mice, maintained through in‐house brother‐sister pairing, were examined at ages prior to the onset of OA (8‐11 weeks, N = 6) and at advanced OA stages (40 weeks, N = 6) [50]. Age‐matched CBA mice (Charles River, UK) served as healthy controls (N = 6 per age) [49, 58]. All mice were kept in polypropylene cages at 21 ± 2°C, subjected to 12 h:12 h light/dark cycles and fed a standard RM1 maintenance diet (No.1; Special Diet Services, Witham, UK) and water ad libitum. All experiments were designed, conducted, and reported in accordance with the ARRIVE guidelines.

Following cervical dislocation, left hindlimbs were removed and skinned, leaving all soft tissues and musculature intact. Whole hindlimbs were individually wrapped in 1X phosphate buffered (PBS) saline‐soaked gauze and frozen at –80°C in sealed polypropylene tubes until in situ loading and sCT. Immediately after imaging, hindlimbs were fixed in 4% neutral buffered formalin (v/v) for 48 h at 4°C, then stored in 70% ethanol (v/v) until further analysis.

### 5.2 In Situ Loading

Frozen hindlimbs were thawed at room temperature and mounted in a Deben CT500 in situ compression stage (Deben UK LTD, UK) equipped with: a 100 N loadcell (accuracy ± 1%), an actuator with a 10 mm compression range and displacement resolution of 3 µm (linearity 1% of full scale), and a custom‐designed open frame comprising an aluminum alloy crossbar (grade 6082, Atomic Precision, UK) bridging two carbon fiber epoxy rods (Ø 6 mm ± 0.2 mm and 120 mm length; Carbon Fibre Profiles, UK) threaded into an aluminum alloy base cover (grade 6082, Atomic Precision, UK). This configuration facilitates precise sample mounting and alignment while enhancing X‐ray transmission efficiency and maximal signal intensity at the detector.

Hindlimbs were secured in a pair of vertically aligned bespoke polyether ether ketone sample holders (Atomic Precision, UK) with the upper cup supporting the flexed knee joint and the lower cup securing the ankle at 45° flexion [51]. Axial compression was applied in the z‐direction upward from the lower cup, prior to the acquisition of sCT images. Given the requirement of maintaining joint stability during sCT acquisition, in situ loading was performed under quasi‐static conditions. A 2 N baseline load was first applied to maintain deep flexion of the knee and ankle [52, 119] with the first series of sCT images acquired following a 15 min stress relaxation period. Three successive sCT scans, each preceded by a 15 min stress‐relaxation period, were acquired following incremental displacement‐controlled loading steps of 20, 70 and 170 µm applied at a motor speed of 0.1 mm/min.

### 5.3 Phase Contrast sCT

sCT images of in situ loaded murine knees were acquired using a filtered white X‐ray beam (2.33 mm aluminum and 0.41 mm copper) with an average energy of 63 keV, on the BM05 beamline at the European Synchrotron Radiation Facility, Grenoble, France, at an isotropic voxel size of 1.45 µm. Incoming filtered X‐rays were magnified with a 5x objective (numerical aperture of 0.21) and detected using a custom‐made 50 µm LuAG:Ce scintillator (Crytur, Czechia) combined with a PCO.edge 4.2 camera (2048 × 2048 pixels, PCO Imaging, Germany). Following the acquisition of 41 flatfield and 40 darkfield images, 6000 projections were acquired in half‐acquisition fly‐scan mode [131], with the center of rotation positioned on the right edge of the image, resulting in a final field of view of 5.21 mm x 2.97 mm. Projections were captured over a continuous 360° rotation with an angular step size of 0.06° and an exposure time of 18 milliseconds. The radiation dose on the samples was estimated to be approximately 7.2 kGy per scan (28.4 kGy per sample). The propagation distance between the sample and detector was iteratively tested before being set to 0.25 m, enabling optimum in‐line phase contrast in sCT images.

Raw sCT projections were flatfield and darkfield corrected with grayscale intensities normalized to the synchrotron current (operated in 200 mA mode) ahead of tomographic reconstruction into 16‐bit TIFF stacks using the in‐house PyHST2 software (version 2023a) [132]. Following normalization, Paganin phase‐retrieval (δ/β = 500) [133] was applied, along with a 2D unsharp mask (σ = 1, coefficient = 3.5) in preparation for filtered back‐projection [134] and ring artifact removal [135].

### 5.4 sCT Image Preprocessing Prior to DVC

Reconstructed sCT datasets were imported into Avizo3D (version 2022.2, Thermo Fisher Scientific, USA) and prepared for global DVC and anatomical segmentation. Image volumes were cropped to the smallest bounding region enclosing the knee joint, with identical crop dimensions applied across all sCT images of the same knee joint following in situ loading. The proximal tibia, comprising the epiphysis and metaphysis, was segmented in cropped 2 N baseline images using a 3D region‐growing algorithm. Osteocyte and chondrocyte lacunae were filled using the “fill volume” function, followed by a 3‐pixel dilation and erosion step before the metaphyseal growth plate was manually removed, resulting in a binary label image of the tibial epiphysis containing growth plate bridges.

Surfaces were created from binarized epiphyseal segmentations (smoothing factor = 5) and simplified by adjusting node spacing prior to the generation of unstructured FE tetrahedral meshes [45]. DVC uncertainty was assessed using sequential repeat reference scans of a single knee joint without additional loading, using a nodal spacing of 30 voxels (43.5 µm) yielding optimal performance (displacement accuracy of 0.012 voxels, precision of 78.3 nm; average strain accuracy of 53 µstrain and precision of 221 µstrain; see Supplementary Information for further details). This mesh size was therefore selected to balance DVC uncertainty with sensitivity to deformation associated with lacunar‐scale microstructural features, including osteocyte lacunae (diameter ∼5–15 µm [136],) and chondrocyte lacunae (diameter ∼25–30 µm [137],). Resulting meshes contained on average 14 229 ± 173 nodes and 53 412 ± 997 tetrahedra per sample for subsequent DVC analyses.

### 5.5 Bulk Epiphyseal Strain Analysis

Prior to DVC analyses, cropped sCT images acquired after the application of 20, 70, and 170 µm displacement were rigidly registered to the corresponding 2 N baseline images of the same sample to correct for translation and rotation. FE tetrahedral meshes derived from tibial epiphysis segmentations were then applied to registered baseline 2 N reference scans and deformed sCT datasets, and displacement fields were incrementally computed using the XDVC module (Avizo3D): 2 N vs. 20 µm (+ 20 µm), 2 N vs. 70 µm (+ 70 µm), and 2 N vs. 170 µm (+ 170 µm). A global FE‐based correlation algorithm was applied, whereby the whole volume of interest is analyzed, and displacement is obtained by minimizing the sum of squared differences between the reference and deformed images, specifically over this region of interest [138]. The derivatives of the displacements with respect to the tetrahedral mesh were used to calculate the deformation gradient and all components of the Green‐Lagrange strain tensors at each nodal location upon algorithm convergence. Two samples per age and genotype were excluded from subsequent DVC analyses as displacement fields could not be reliably correlated due to mechanical failure of the tibial epiphysis during in situ loading; these samples were retained for subsequent µCT‐based tibial morphometric analyses. Point clouds extracted from DVC FE‐mesh nodes following stepwise correlations were thresholded to remove strains above 10 000 µstrain (1%) in line with published literature [114, 139, 140, 141], with 11 964 ± 602 nodes per epiphysis retained for distributional analyses. Scientific color maps were used for strain visualization, with “Navia” [142] and “Matter” [143] applied to compressive and tensile strains, respectively, to minimize visual distortion and ensure accessibility for readers with color‐vision deficiencies [144].

### 5.6 Anatomical Epiphyseal Characterization

Cortical and trabecular compartments of tibial epiphyses were segmented in cropped 2 N baseline sCT images using a method adapted from Herbst et al., 2021 [145] in Avizo3D. Adjustments for higher resolution images included the removal of a non‐local means filter (as Paganin phase‐retrieval was already applied) and increasing ball erosion/dilation operations to 9‐pixels and ball‐closing operation to 75‐pixels. Briefly, the medial and lateral SCP were manually segmented in the coronal plane in 29 µm increments, interpolated and corrected as required prior to subtraction from the total cortex using the “arithmetic” function yielding segmentations of the ECC. SCP pores were isolated by a 2‐pixel erosion and manual thresholding, then subjected to a single pixel erosion/dilation step. Extracted pores from individual epiphyses were exported as binary .tiff image stacks and subjected to 3D individual object analysis in CTAn (version 1.23.0.2 +; Bruker, Belgium). Pores smaller than 20 µm3 were classified as noise in line with the published literature [146, 147, 148]. Condylar trabecular bone was manually subdivided into lateral and medial compartments using the “lasso” tool; these volumes were then subtracted from the total trabecular segmentation, enabling isolation of the ECTB compartment. Local thickness maps were generated from these compartment segmentations in Avizo3D and represent relative compartment‐specific thickness values. Compared with lower‐resolution µCT‐based epiphyseal analyses, higher‐resolution phase‐contrast sCT can resolve finer structural features, which may contribute to lower apparent local thickness values. Thickness values derived from these maps were therefore interpreted comparatively rather than as absolute structural measurements.

To isolate compartment‐specific DVC‐derived strains, volumetric bulk strains were imported into Avizo3D, spatially aligned, and resampled to match the dimensions of the sCT datasets and segmentations described above. The resampled volume was then subjected to interactive thresholding and masking, performed to isolate the relevant anatomical structure, containing DVC‐derived strains, while removing the background in preparation for further analysis.

### 5.7 Spatial Registration between DVC Point Cloud and FE Model Nodes

DVC point clouds were registered to FE model nodes using bespoke code in MATLAB (v. R2023b, MathWorks, Natick, US). A new DVC mesh (mesh 2) with identical node number and spatial positions was generated and aligned to the FE model mesh (mesh 3). Principal strain values from the original DVC mesh (mesh 1) were mapped to mesh 2 using natural‐neighbor interpolation from nodes within the mesh 1 domain and nearest‐neighbor extrapolation for nodes outside of it. DVC‐FE comparisons were performed at matched nodal locations after resampling DVC strain values onto mesh 3, rather than on an element‐by‐element basis. Multiple DVC points within a single FE element were therefore not directly averaged.

### 5.8 Material Property Optimization and FE Modeling

FE models of individual CBA and STR/Ort tibial epiphyses were generated from anatomical segmentations in Avizo3D. Binary segmentation labels were converted into surfaces before a convergence test was performed to identify an optimal node spacing of 25 voxels (36.3 µm; <1% strain between refinements), with each node assigned a homogeneous elastic modulus of 150 MPa and Poisson's ratio of 0.3. Converged meshes were exported in the INP format for further optimization (Figure S5A). Each segmented mineralized compartment of the tibial epiphysis was assigned to a homogeneous, linear elastic, isotropic material model. This simplified constitutive representation was used to define an effective compartment‐specific mechanical response under the applied experimental loading conditions and to enable comparison of regional load distribution patterns across genotypes, ages, and anatomical compartments. The model was not intended to capture non‐linear, anisotropic, or time‐dependent tissue behavior.

Idealized boundary conditions were applied to define a controlled inverse FE problem representing the experimental loading configuration. External epiphyseal nodes corresponding to the superior condylar surface were fully constrained (set‐1), while uniaxial compressive displacements of 20, 70, and 170 µm were applied along the z‐axis to inferior epiphyseal nodes (set‐2), matching the displacement increments imposed during in situ loading. The FE model did not explicitly include cartilage, meniscus, ligaments, or other periarticular tissues; therefore, these boundary conditions were used to enable direct comparison with DVC‐derived strain fields within the mineralized tibial epiphysis, rather than to reconstruct whole‐joint physiological load sharing. As such, the resulting material parameters should be interpreted as effective regional properties rather than intrinsic tissue‐level constants. FE simulations were conducted using Abaqus (version 2023, Simulia, Providence, USA) with the implicit solver to compute strain fields within each anatomical region of the tibial epiphyses.

Material properties for the six anatomical compartments of the tibial epiphysis were estimated using an inverse optimization framework implemented in MATLAB (Zenodo https://doi.org/10.5281/zenodo.15704567), varying only the elastic modulus (10–12 000 MPa) and Poisson's ratio (0.1–0.45) while geometrical boundary conditions and loading remained fixed. The simulated first and third principal strain fields were compared to spatially registered DVC‐derived strains, with spatial correspondence evaluated following the calculation of the root‐mean‐square error (RMSE, Figure S5B,C). The MATLAB Global Optimization Toolbox was used to iteratively adjust the material properties to achieve the global minimum RMSE, with the final regional material properties retained for further analysis.

### 5.9 Bland–Altman Plot

Bland–Altman plots were used to assess agreement between the FE‐predicted and DVC‐derived strains. Material properties were optimized using the first principal strain generated following 70 µm displacement, while first principal strain generated in response to + 20 µm and + 170 µm displacement, along with the third principal strain arising in response to + 20 µm, + 70 µm, and + 170 µm displacement, were used for validation. Bland–Altman plots were generated in OriginPro (version 2023b, Massachusetts, USA; Figure S5D,E) by calculating the mean and difference between FE‐predicted and DVC‐derived strain data. The 95% limits of agreement were defined as the mean ± 1.96 × SD.

### 5.10 µCT and Analysis

After in situ testing and sCT, whole hindlimbs were imaged by µCT using a Skyscan 1172 scanner (Bruker, Belgium) (N = 6 per age and genotype) at an isotropic voxel size of 4.98 µm, with the X‐ray tube operated at 50 kV and 200 µA, using an exposure time of 960 milliseconds and a 0.5 mm aluminum filter. Projections were acquired every 0.6 ° over a continuous rotation of 180° and reconstructed using NRecon (version 1.7.4.6; Bruker, Belgium). Hydroxyapatite phantoms (0.25 and 0.75 g/cm^3^) (Bruker, Belgium) were scanned and reconstructed under identical conditions for attenuation coefficient calibration.

Reconstructed µCT images were reoriented in Dataviewer (version 1.5.6.2; Bruker, Belgium) prior to segmentation in CTAn. Epiphyseal analyses used regions proximal to the unmineralized growth plate. For metaphyseal trabecular bone analyses, 5% of the total bone length, beginning where the primary spongiosa disappears, was used. Datasets were manually segmented and binarized using a threshold of 85 for epiphyseal and metaphyseal morphometric analyses [147, 149]. Diaphyseal cortical bone morphometry was analyzed on a slice‐by‐slice basis following manual segmentation and binarization using a threshold of 80, excluding the proximal and distal 10% of the tibial length. 3D renderings were produced in Avizo3D.

Growth plate bridges were analyzed as previously described [120]. Briefly, reoriented µCT datasets were imported into Avizo3D, and tibiae were segmented using a 3D region‐growing algorithm. Growth plate bridges centered across the width of the growth plate were identified across all planes. A bespoke in‐house python script implemented in Avizo3D was used to quantify bridge number and spatial distribution prior to projection onto the tibial epiphysis and pseudo‐colored using the “Batlow” [142] scientific color map to reflect the areal density at the bridge location.

### 5.11 Histology and OARSI Scoring

Following µCT, joints were decalcified in 10% ethylenediaminetetraacetic acid (EDTA) at 4°C for 14 days prior to being processed for paraffin wax embedding, conducted in the frontal orientation. Serial coronal sections of 6 µm thickness were cut throughout each knee joint, with every fourth slide (approximately 8–10 sections per joint) stained with Safranin‐O (0.5% w/v in dH_2_O) and counterstained with Fast Green (0.2% w/v in dH_2_O). Articular cartilage lesions across lateral and medial tibial condyles were blindly scored using the OARSI scoring system [150] by three independent scorers (AS, LAEE, LEB), with both maximum and average scores recorded for each condyle. Sections that could not be reliably graded due to section loss, inadequate section quality or poor preservation of the articular surface were excluded from analysis. Inter‐scorer agreement was considered acceptable if the intraclass correlation coefficient was 0.88 (95% confidence interval: 0.65–0.95).

### 5.12 Statistical Analysis

All investigators were blinded to the genotype and age of the animals throughout experimentation until data analysis. Unless otherwise stated, data from individual animals (N = 4–6 per age and genotype) are presented as box and whisker plots. For distributional analyses of nodal DVC‐derived strains, data are presented as violin plots with each violin representing pooled nodal strain values for each experimental group derived from individual animals (N = 4 per age and genotype). For 2D cortical morphometric analyses, data from individual animals (N = 6 per age and genotype) are presented as line graphs showing mean ± standard error of the mean (SEM), and were generated in RStudio (version 2024.12.0, build 467; R Foundation for Statistical Computing, Austria). Summary data presented in tables are reported as mean ± standard deviation (SD).

Statistical analyses were performed on animal‐level summary values in GraphPad Prism (version 10.5.0; Massachusetts, USA) unless otherwise stated. Normality was assessed using the Shapiro–Wilk test, and no significant deviations from normality were detected. Differences in pooled DVC‐derived strain distributions between genotypes and age groups were evaluated using the Kolmogorov‐Smirnov test. One‐way ANOVA was used to assess the effect of increasing displacement on strain within genotypes and age groups relative to the + 20 µm displacement step. Two‐way ANOVA was used to determine the independent and interactive effects of genotype and age on compartmentalized epiphyseal strain, epiphyseal anatomy, FE‐derived modulus and Poisson's ratio, growth plate bridge number and areal density, and trabecular bone morphometric parameters. Linear mixed‐effects models were used to assess the effects of genotype, age, condyle, and their interactions on OARSI score, with mouse ID included as a random effect to account for paired lateral and medial condyle scores from the same joint. Šídák's post hoc test was conducted for further comparisons. For cortical bone morphometric analyses, two‐way ANOVA was performed using bespoke code implemented in RStudio, with region‐matched significance displayed in colored heatmaps. A *p*‐value of <0.05 was considered statistically significant.

## Author Contributions

**Aikta Sharma**: methodology, software, data curation, investigation, validation, formal analysis, visualization, writing – original draft, writing – review and editing, conceptualization.**Lucinda A.E. Evans**: methodology, validation, writing – original draft, writing – review & editing, formal analysis, software, data curation, visualization. **Lucie E. Bourne**: software, formal analysis, data curation, writing – original draft, methodology, validation, visualization. **Jishizhan Chen**: methodology, software, data curation, formal analysis, writing – original draft, writing – review & editing, validation. **Alissa L. Parmenter**: methodology, sofrware, investigation, formal analysis, writing – original draft, visualization. **Joseph Brunet**: software, data curation, writing – original draft, methodology, investigation, visualization, resources. **Kamel Madi**: writing – original draft, visualization, validation, methodology, software, resources. **Sebastian Marussi**: methodology. **Andrew A. Pitsillides**: conceptualization, funding acquisition, writing – original draft, writing – review & editing, supervision, resources, project administration, visualization, methodology. **Peter D. Lee**: conceptualization, methodology, supervision, funding acquisition, project administration, writing – original draft, writing – review & editing, visualization. **Katherine A. Staines**: conceptualization, funding acquisition, writing – original draft, writing – review & editing, visualization, methodology, project administration, resources, supervision.

## Funding

This work was supported by the UKRI MRC (MR/V033506/1), the BLAST Network (BB/W01825X/1), the Royal Academy of Engineering (CiET 1819/10) and the Chan Zuckerberg Initiative (CZIF2021‐006424 and CZIF2022‐316777).

## Conflicts of Interest

The authors declare no conflicts of interest.

## Supporting information




**Supporting File 1**: advs76716‐sup‐0001‐SuppMat.docx.

**Supporting File 2**: advs76716‐sup‐0002‐TableS1‐S3.zip.

## Data Availability

The processed sCT datasets, full force‐displacement‐time curves, DVC‐derived strain fields, and FE models are available from the corresponding authors upon reasonable request.
