# Supplementary material for: In Situ Profiling of Nanoscale Strains Uncovers Mechano‐Architectural Predictors of Aging and Osteoarthritis Emergence
Source: Adv Sci (Weinh). 2026 Jul 23:e76716. Online ahead of print. doi: 10.1002/advs.76716 (PMC13395401; doi:10.1002/advs.76716)
Supplement: Supplementary file 1 — Supporting File 1: advs76716‐sup‐0001‐SuppMat.docx. [file ADVS-9999-e76716-s002.docx]

Supporting Information

In situ profiling of nanoscale strains uncovers mechano-architectural predictors of aging and osteoarthritis emergence

*Aikta Sharma*, Lucinda AE Evans, Lucie E Bourne, Jishizhan Chen, Alissa L Parmenter, Joseph Brunet, Kamel Madi, Sebastian Marussi, Andrew A Pitsillides*, Peter D Lee* & Katherine A Staines**

**DVC Uncertainty Measurement Results**

To determine the appropriate finite element tetrahedral mesh size for DVC analyses and for the evaluation of DVC performance, both systematic and random displacement error uncertainties and strain precision were determined using the DVC uncertainty algorithm in Avizo3D (XDVC module). Tibial epiphyses from repeat sCT scans of a separate STR/Ort mouse knee joint, which was not included in the study cohort or in the analyses reported in the main manuscript, were segmented as described in the methods and used to generate a series of FE tetrahedral meshes with varying minimum distances between nodes. A mesh size with a minimum node spacing of 30 voxels (43.5 µm) was identified as optimal, yielding mean displacement errors (referred to as accuracy) of 0.458 (x), 0.621 (y), and 0.0122 (z) voxels, corresponding to 0.665, 0.901 and 0.018 µm (x, y and z, respectively). DVC precision, quantified as the standard deviation of the displacement field, was 0.012 (x), 0.043 (y), and 0.054 (z) voxels, which corresponded to 0.017, 0.063 and 0.079 µm (x, y and z, respectively). Similarly, average strain accuracy was 53 µstrain (E_xx_=65 µstrain, E_yy_=95 µstrain, E_zz_=108 µstrain, E_xy_=25 µstrain, E_yz_=29 µstrain and E_xz_=16 µstrain) while average strain precision was 221 µstrain (E_xx_=233 µstrain, E_yy_=181 µstrain, E_zz_=415 µstrain, E_xy_=127 µstrain, E_yz_=178 µstrain and E_xz_=196 µstrain).

**Supplementary Figures**


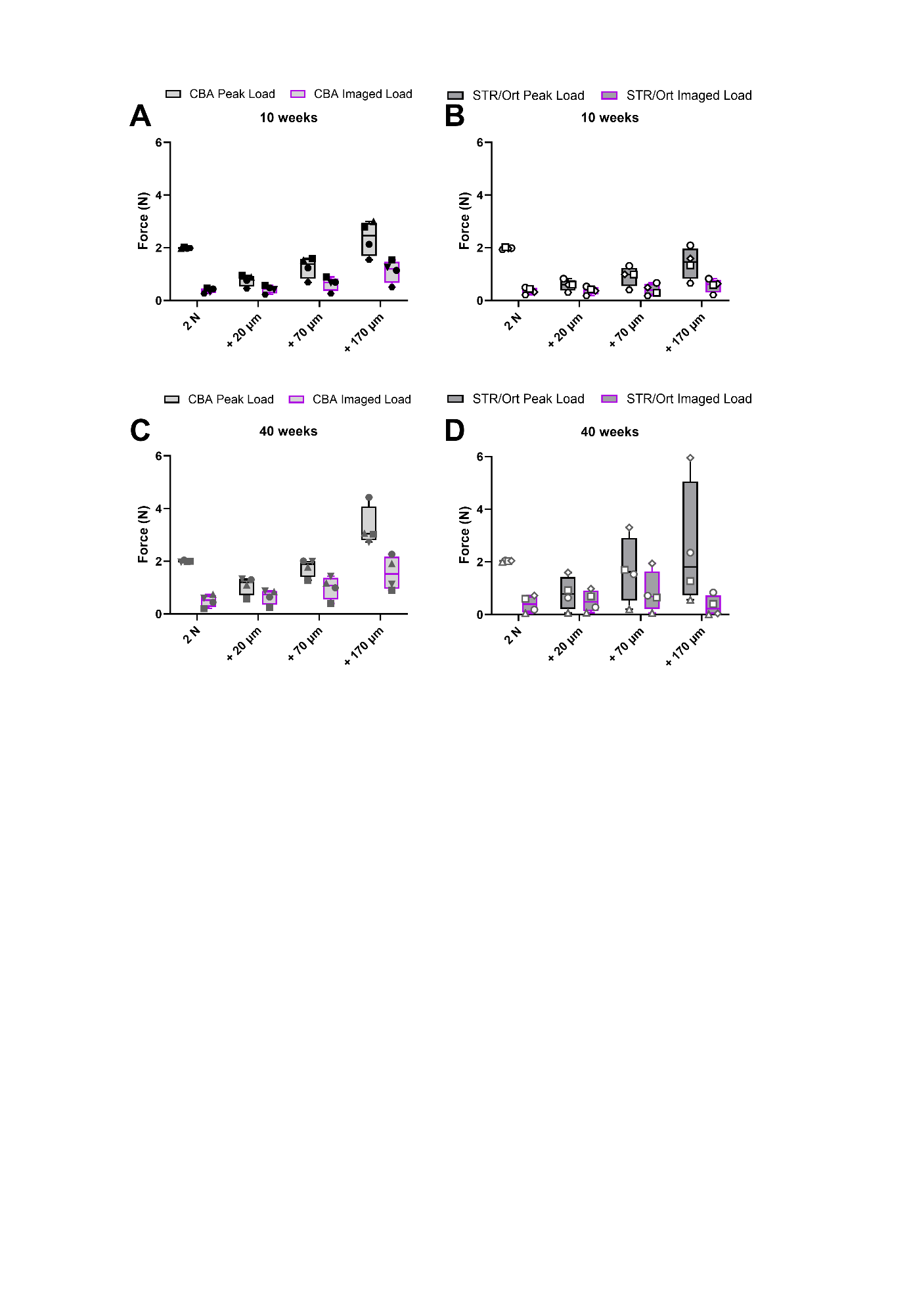


**Supplementary Figure S1. Loading regime for in situ compression of 10- (A) and 40-week-old CBA (B) and 10- (C) and 40-week-old STR/Ort (D) knee joints.** Peak forces (black outlined boxes) were recorded following a 2 N baseline load and after sequential displacement increments of 20 µm, 70 µm, and 170 µm. sCT images were acquired after a 15 minute stress-relaxation period, once peak loads had relaxed and stabilized (hereafter referred to as imaged loads; purple outlined boxes). Data are presented as box and whisker plots, where boxes represent the interquartile range (IQR), the central line denotes the median, and whiskers correspond to the minimum and maximum values. Symbols represent individual animals (N=4 per age and genotype).


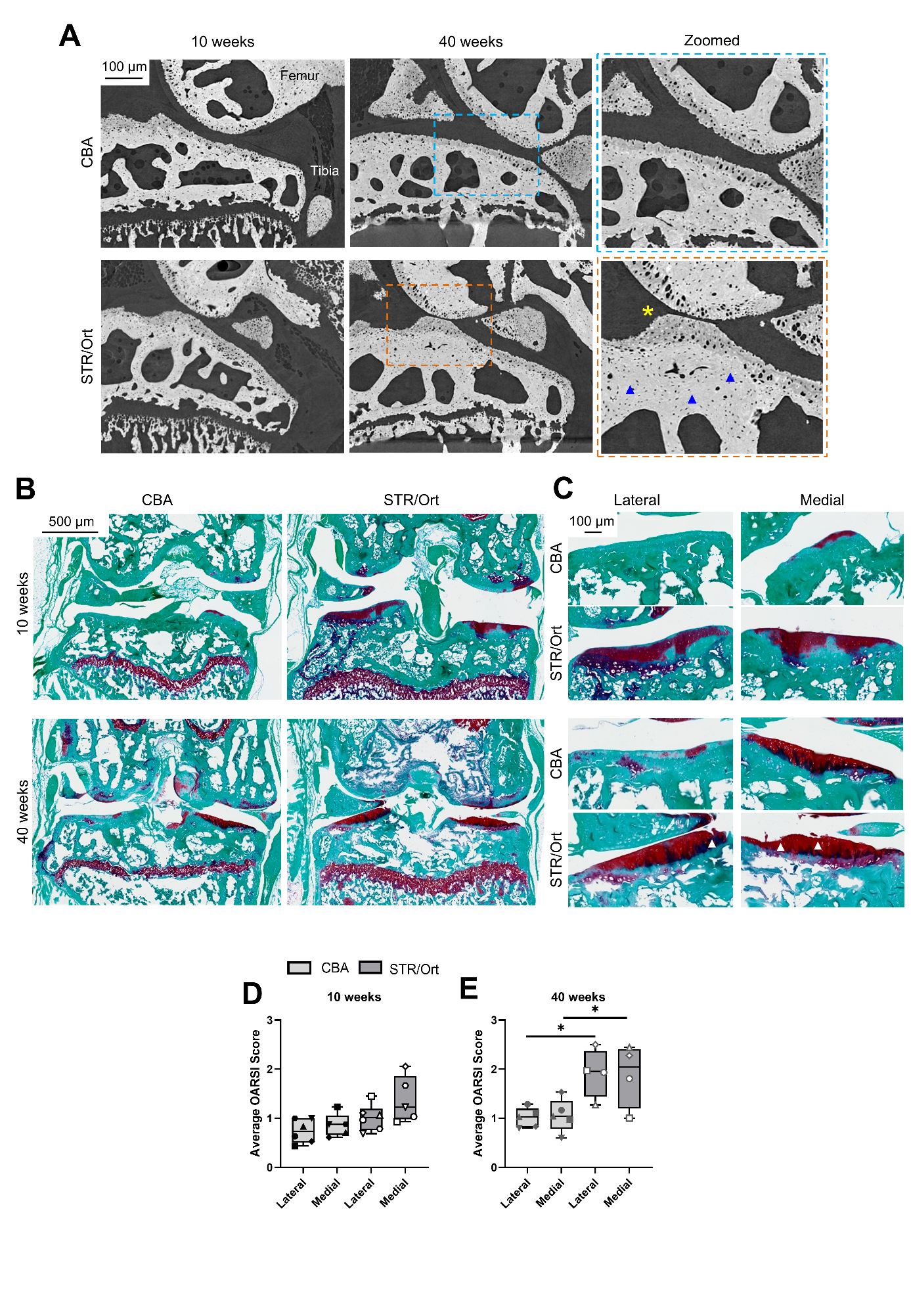


**Supplementary Figure S2.** sCT visualization of epiphyseal microstructure highlights age-associated osteoarthritic pathology within the tibial epiphyses of STR/Ort knee joints. CBA knee joint architecture is preserved with age (top panel) and is comparable to that observed in 10-week-old STR/Ort mice (bottom panel) (A, scale bar=100 µm). By 40 weeks of age, advanced osteoarthritic changes are evident in STR/Ort knee joints (middle bottom, orange hatched box), including joint space narrowing (yellow asterisk) and subchondral thickening (blue arrowheads), which are not evident in age-matched CBA mice (blue hatched box). Histological examination of decalcified, paraffin-embedded knee joints following sCT and Safranin-O/Fast-Green staining (B, scale bar=500 µm) highlights age-associated cartilage lesions in the lateral and medial condyles of STR/Ort mice (C, white arrowheads; scale bar=100 µm). Average OARSI scores of the articular cartilage across lateral and medial tibial condyles were comparable between genotypes at 10 weeks of age (D) whereas greater average scores were evident in STR/Ort mice at 40 weeks of age (E) compared to CBA mice. Data are presented as box and whisker plots, where boxes represent the IQR, the central line denotes the median, and whiskers correspond to the minimum and maximum average value. Symbols represent individual animals (N=4-6 per age and genotype). Statistical significance between condyles was assessed using linear mixed-effects models with Šídák’s post hoc test (*p<0.05).

**Supplementary Table S1.** Median and IQR of compressive strains in the tibial epiphyses of CBA and STR/Ort mice at 10 and 40 weeks of age following applied in situ displacements of + 20, + 70 and + 170 µm relative to 2 N baseline loads. Values are derived from N=4 mice per age and genotype.

|  |  | | **CBA** | **STR/Ort** |
| --- | --- | --- | --- | --- |
| **10 weeks** | + 20 µm | Median | –0.0009 | -0.0012 |
|  |  | IQR | 0.0016 | 0.0017 |
|  | + 70 µm | Median | -0.0013 | -0.0014 |
|  |  | IQR | 0.0019 | 0.0022 |
|  | + 170 µm | Median | -0.0016 | -0.0017 |
|  |  | IQR | 0.0022 | 0.0023 |
| **40 weeks** | + 20 µm | Median | –0.001 | -0.0013 |
|  |  | IQR | 0.0017 | 0.0019 |
|  | + 70 µm | Median | –0.0012 | -0.0013 |
|  |  | IQR | 0.0018 | 0.0019 |
|  | + 170 µm | Median | –0.0013 | -0.002 |
|  |  | IQR | 0.002 | 0.003 |

**
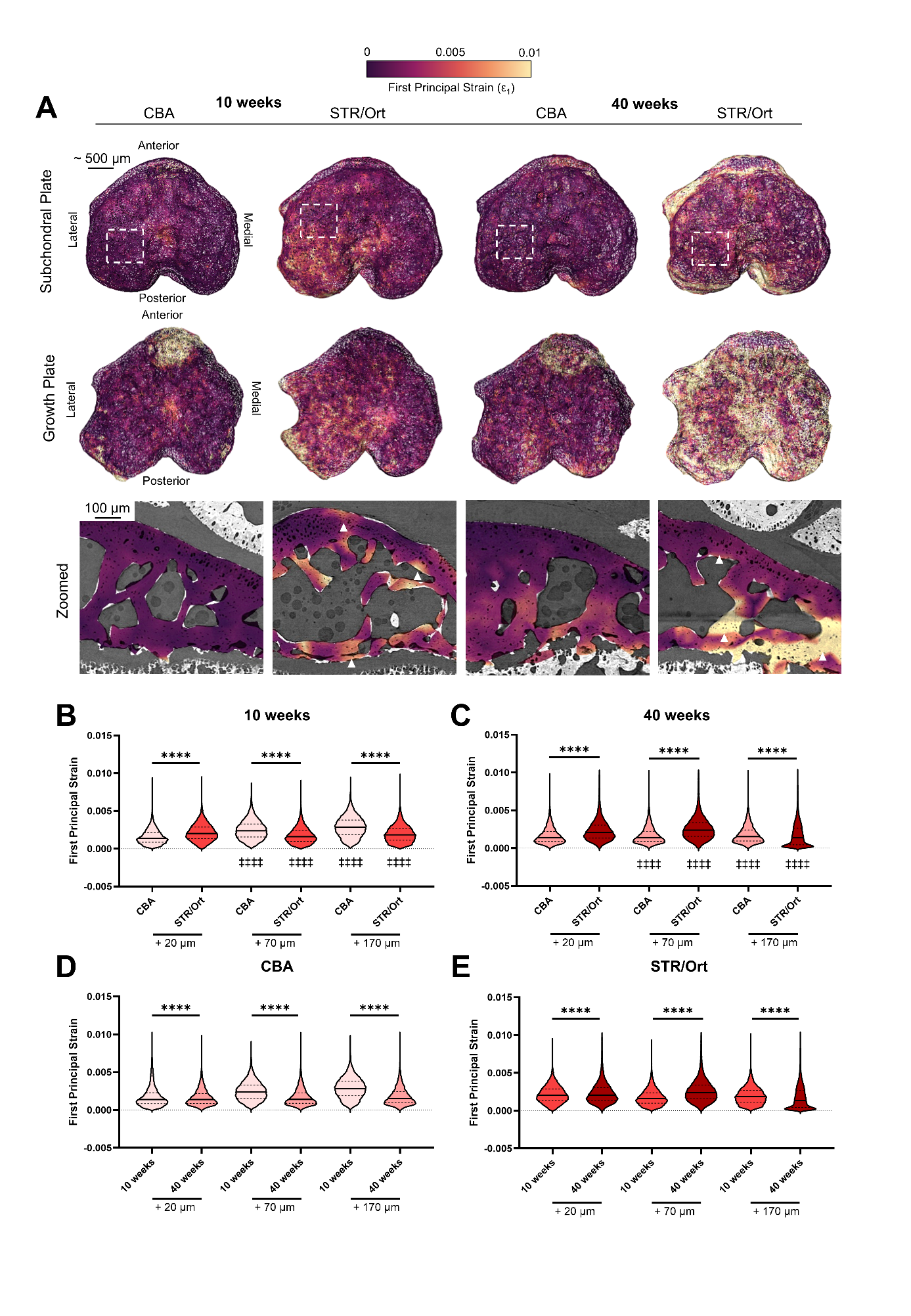
**

**Supplementary Figure S3.** DVC-computed tensile strains superimposed on FE tetrahedral meshes of 10- and 40-week-old CBA and STR/Ort tibial epiphyses, viewed from the SCP (top row) and growth plate (bottom row), showing localisation of high (pale yellow) and low (dark purple) first principal strain. In CBA mice, high magnitude tensile strains are predominantly confined to growth plate regions in epiphyses of both ages. In STR/Ort epiphyses, high magnitude tensile strains are evident within regions of the SCP and growth plate and increase with age (white arrowheads). Zoomed sagittal sCT images overlaid with tensile strain maps show distal strain concentration in CBA epiphyses at both ages, whereas higher magnitude strains are distributed across the tibial epiphyses of STR/Ort mice. Tensile strain distributions from DVC FE-mesh nodes in response to displacement-induced loading are presented as violin plots for 10-week-old (B) and 40-week-old (C) CBA and STR/Ort epiphyses. The effect of age on tensile distribution is shown for CBA (D) and STR/Ort (E) epiphyses in response to incremental displacement. Individual violins represent pooled DVC-derived strains from N=4 mice per age and genotype; the solid line indicates the median, and dashed lines indicate the 25^th^ and 75^th^ percentiles. Statistical significance between pooled DVC-derived strain distributions in CBA and STR/Ort mice was assessed using the Kolmogorov-Smirnov test (**p<0.01, ***p<0.001 and ****p<0.0001). Statistical significance between displacement-induced load steps relative to the + 20 µm step was assessed using one-way ANOVA with Šídák’s post hoc test (‡‡‡‡p<0.0001).

**Supplementary Table S2.** Median and IQR of tensile strains in the tibial epiphyses of CBA and STR/Ort mice at 10 and 40 weeks of age following applied in situ displacements of + 20, + 70 and + 170 µm relative to 2 N baseline loads. Values are derived from N=4 mice per age and genotype.

|  |  | | **CBA** | **STR/Ort** |
| --- | --- | --- | --- | --- |
| **10 weeks** | + 20 µm | Median | 0.0012 | 0.0019 |
|  |  | IQR | 0.0014 | 0.0021 |
|  | + 70 µm | Median | 0.0022 | 0.0015 |
|  |  | IQR | 0.0026 | 0.0017 |
|  | + 170 µm | Median | 0.0029 | 0.0017 |
|  |  | IQR | 0.0028 | 0.002 |
| **40 weeks** | + 20 µm | Median | 0.0011 | 0.0015 |
|  |  | IQR | 0.0019 | 0.0016 |
|  | + 70 µm | Median | 0.0012 | 0.0018 |
|  |  | IQR | 0.0016 | 0.0016 |
|  | + 170 µm | Median | 0.0013 | 0.0018 |
|  |  | IQR | 0.0025 | 0.0012 |


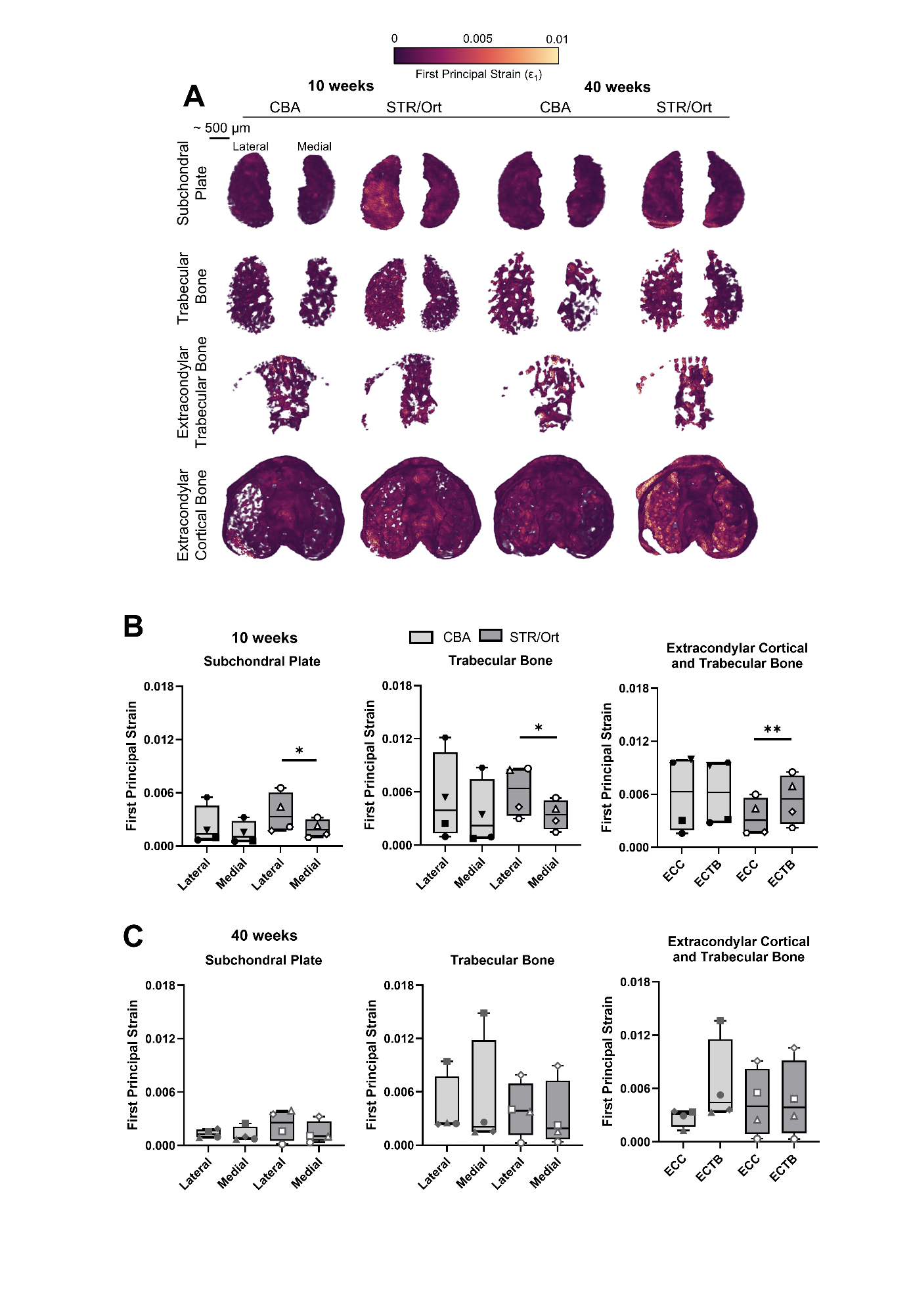


**Supplementary Figure S4.** Regional and condylar DVC-derived tensile strains in the SCP, trabecular bone, ECTB, and ECC of 10- and 40-week-old CBA and STR/Ort tibial epiphyses (A, scale bar=500 µm). Quantification of average tensile strain in the lateral and medial condylar compartments and extracondylar regions is shown for 10-week-old (B) and 40-week-old (C) CBA and STR/Ort mice. At 10 weeks of age, STR/Ort mice show compartment-specific tensile strain differences, including greater lateral than medial strain within the SCP and trabecular bone, and greater strain in the ECTB than ECC. In CBA epiphyses, tensile strains are evenly distributed across lateral and medial condylar compartments. The regional tensile strain differences evident in 10-week-old STR/Ort mice are lost with age. Data are presented as box and whisker plots, where boxes represent the IQR, the central line denotes the median and whiskers correspond to the minimum and maximum values. Symbols represent individual animals (N=4 per age and genotype). Statistical significance between regions was assessed using two-way ANOVA with Šídák’s post hoc test (*p<0.05, **p<0.01).


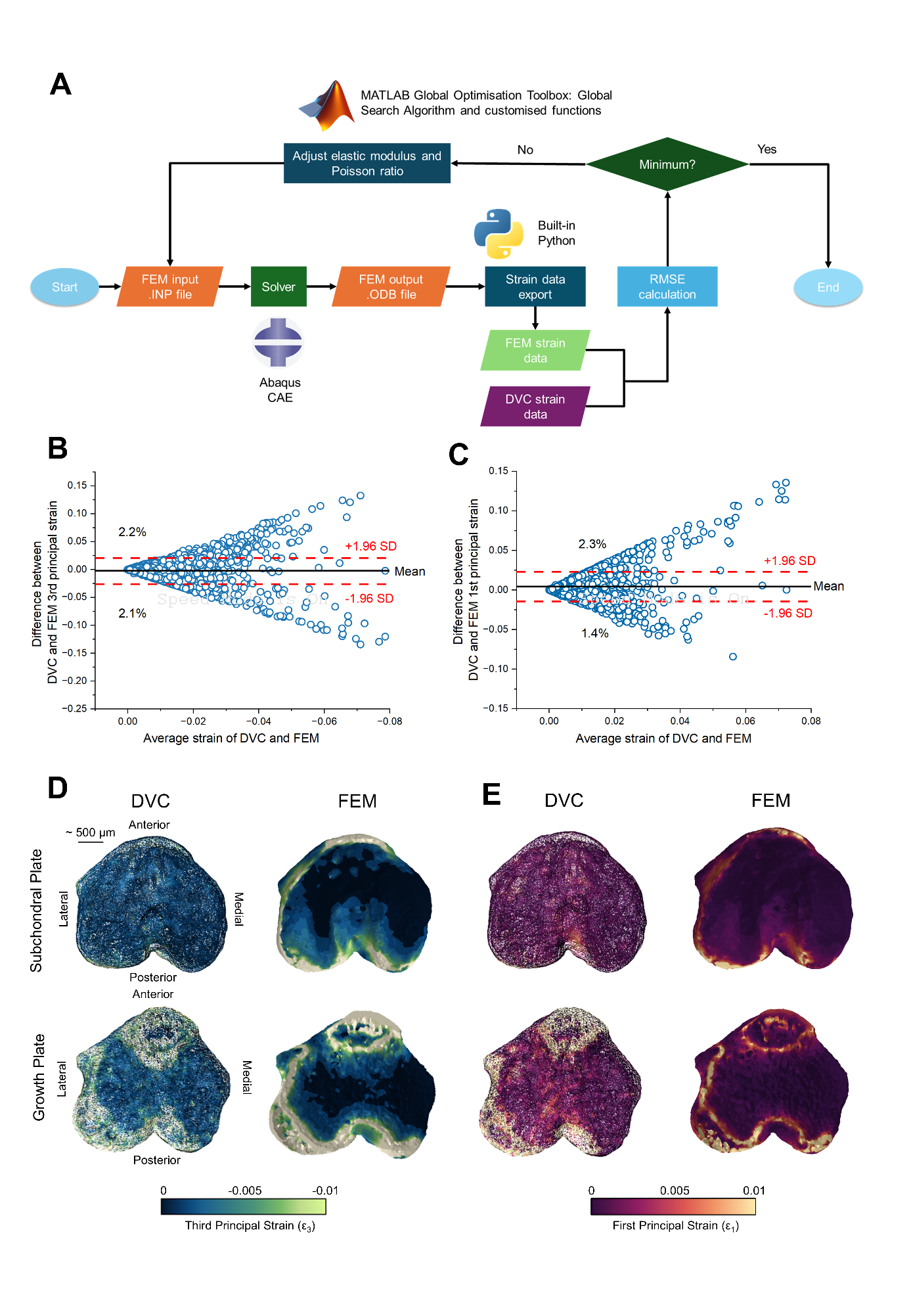


**Supplementary Figure S5.** FE model optimization workflow and validation against DVC-derived strain measurements. Schematic of the iterative optimization protocol used to refine FE-derived material properties (A). Elastic modulus and Poisson’s ratio were systematically adjusted in MATLAB to minimize the RMSE between experimentally measured DVC strain fields and FE-predicted strain data. Bland-Altman plots compare DVC- and FE-derived third principal (compressive, B) and first principal (tensile, C) strains. Representative comparisons of DVC-derived and DVC-optimized FE-derived compressive (D) and tensile (E) strain fields are shown from the SCP (top row) and growth plate (bottom row). DVC-FE comparisons were performed at matched nodal locations after resampling DVC-derived strain values onto the FE nodal reference mesh.

**Supplementary Table S3.** Quantification of regional thickness and volume in the tibial epiphyses of 10- and 40-week-old CBA and STR/Ort mice. Data are presented as mean ± SD for N=4 mice per age and genotype. Statistical significance was assessed using two-way ANOVA with Šídák’s post hoc test. † denotes differences between 10- and 40-week-old animals, * denotes differences between CBA and STR/Ort mice, and § denotes differences between epiphyseal condyles/regions; single, double, triple, and quadruple symbols correspond to p<0.05, p<0.01, p<0.001, and p<0.0001, respectively.

|  |  |  | **Lateral**  **Subchondral Plate** | **Medial**  **Subchondral Plate** | **Lateral**  **Trabecular Bone** | **Medial**  **Trabecular Bone** | **Extracondylar Cortical**  **Bone** | **Extracondylar Trabecular**  **Bone** |
| --- | --- | --- | --- | --- | --- | --- | --- | --- |
| **Thickness (µm)** | **10 weeks** | CBA | 73.06 ± 6.7 | 79.14 ± 3.13 | 34.55 ± 1.42 | 34.72 ± 2.48 | 64.34 ± 3.95 | 28.8 ± 2.8  §§§§ |
|  |  | STR/Ort | 65.07 ± 7.48 | 83.52 ± 8.99  §§§ | 33. 42 ± 3.09 | 37.73 ± 2.05  §§ | 72.95 ± 3.38  ** | 31.01 ± 3.64  §§§§ |
|  | **40 weeks** | CBA | 82.03 ± 6.38 | 93.38 ± 11.58  † | 37.47 ± 1.84 | 36.35 ± 2.01 | 80.31 ± 3.88  †††† | 31.89 ± 1.69  §§§§ |
|  |  | STR/Ort | 79.01 ± 12.95 | 101.14 ± 4.94  §, † | 37.95 ± 3.56 | 36.36 ± 6.23 | 85.11 ± 9.14  † | 33.72 ± 1.67  §§§§ |
| **Volume (mm^3^)** | **10 weeks** | CBA | 0.22 ± 0.01 | 0.16 ± 0.008  §§§§ | 0.18 ± 0.02 | 0.09 ± 0.004  §§§ | 0.95 ± 0.13 | 0.14 ± 0.03  §§§§ |
|  |  | STR/Ort | 0.19 ± 0.02  * | 0.2 ± 0.02  §§ , * | 0.2 ± 0.03 | 0.13 ± 0.01  §§, * | 0.99 ± 0.13 | 0.15 ± 0.01  §§§§ |
|  | **40 weeks** | CBA | 0.23 ± 0.07 | 0.28 ± 0.02  †† | 0.13 ± 0.03  †† | 0.07 ± 0.01  § | 1.28 ± 0.16  †† | 0.09 ± 0.01  §§§§ |
|  |  | STR/Ort | 0.26 ± 0.03  †† | 0.21 ± 0.03  §, * | 0.13 ± 0.02  †† | 0.09 ± 0.03 | 1.2 ± 0.13  † | 0.09 ± 0.008  §§§§ |


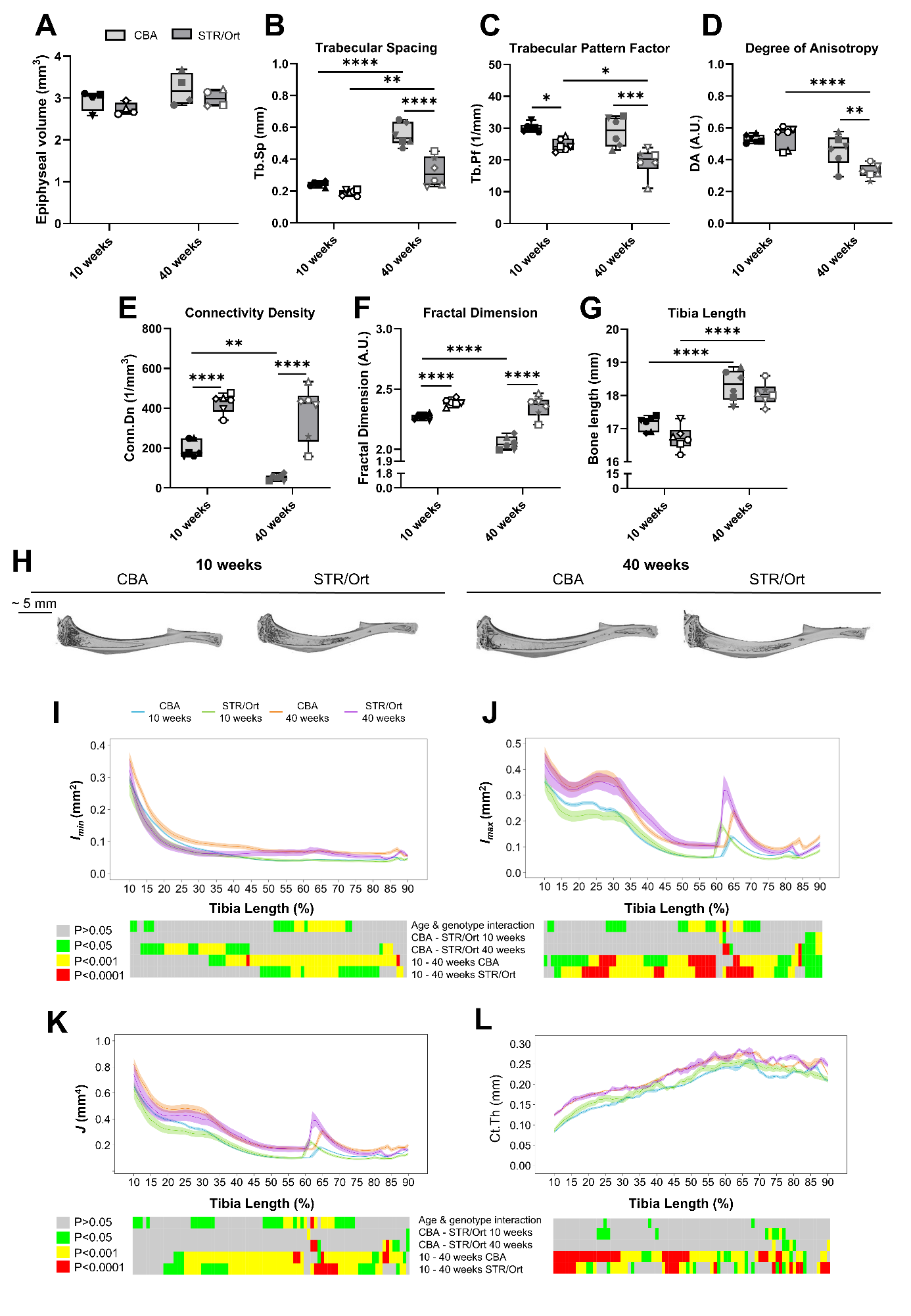


**Supplementary Figure S6.** µCT-based quantification of tibial epiphyseal volume (A), metaphyseal trabecular morphometric parameters, including trabecular spacing (Tb.Sp, B), trabecular pattern factor (Tb.Pf, C), degree of anisotropy (DA, D), connectivity density (Conn.D, E), fractal dimension (F), and tibial bone length (G), in 10- and 40-week-old CBA and STR/Ort mice. Data are presented as box and whisker plots (A-G), where boxes represent the IQR, the central line denotes the median, and whiskers correspond to the minimum and maximum values. Symbols represent individual animals (N=4 per age and genotype, A; N=6 per age and genotype, B-G), and statistical significance was assessed using two-way ANOVA with Šídák’s post hoc test (*p<0.05, **p<0.01, ***p<0.001, and ****p<0.0001). 3D renderings of tibial cortical bone from reconstructed µCT datasets show comparable gross anatomy between CBA and STR/Ort mice at both ages (H). Cortical morphometric analyses of minimum second moment of area (I_min_, I), maximum second moment of area (I_max_, J), polar moment of inertia (*J*, K), and cortical thickness (Ct.Th, L), evaluated between 10-90% of tibial length, are presented as line graphs showing mean ± SEM. Heatmaps beneath each plot show statistical significance from spatially matched locations along the tibial length with age (10 versus 40 weeks) and between genotypes (CBA versus STR/Ort; N=6 mice per age and genotype). Statistical significance was assessed using two-way ANOVA with Tukey’s post hoc test (I-L; gray, p>0.05; p<0.05, green; yellow, p<0.001; red, p<0.0001).
