# Supplementary figures and images for: In Situ Profiling of Nanoscale Strains Uncovers Mechano‐Architectural Predictors of Aging and Osteoarthritis Emergence
Source: Adv Sci (Weinh). 2026 Jul 23:e76716. Online ahead of print. doi: 10.1002/advs.76716 (PMC13395401; doi:10.1002/advs.76716)

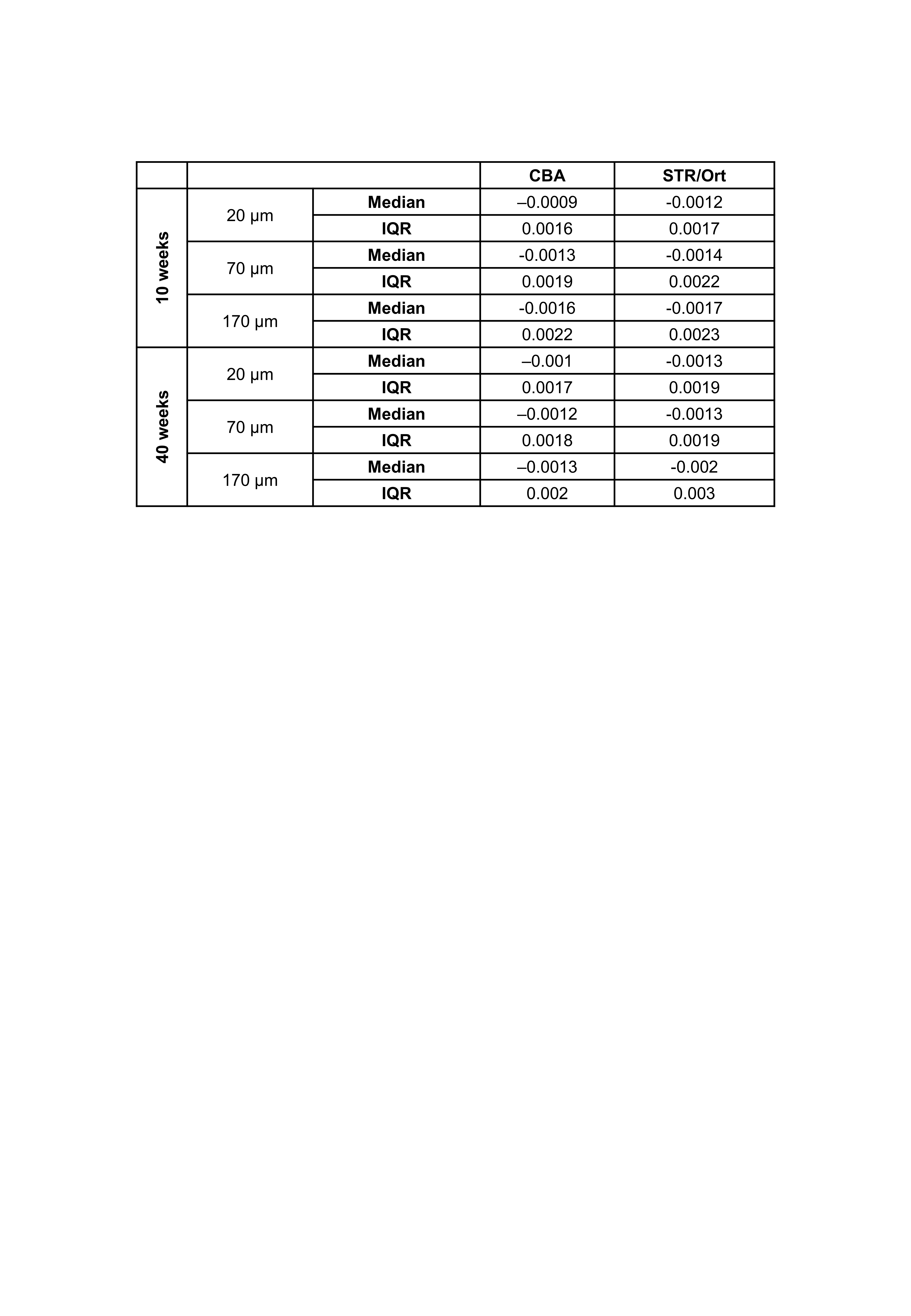

Supplement: Supplementary file 2 — Supporting File 2: advs76716‐sup‐0002‐TableS1‐S3.zip. [file ADVS-9999-e76716-s001.zip › Supplementary Table 1.TIF]

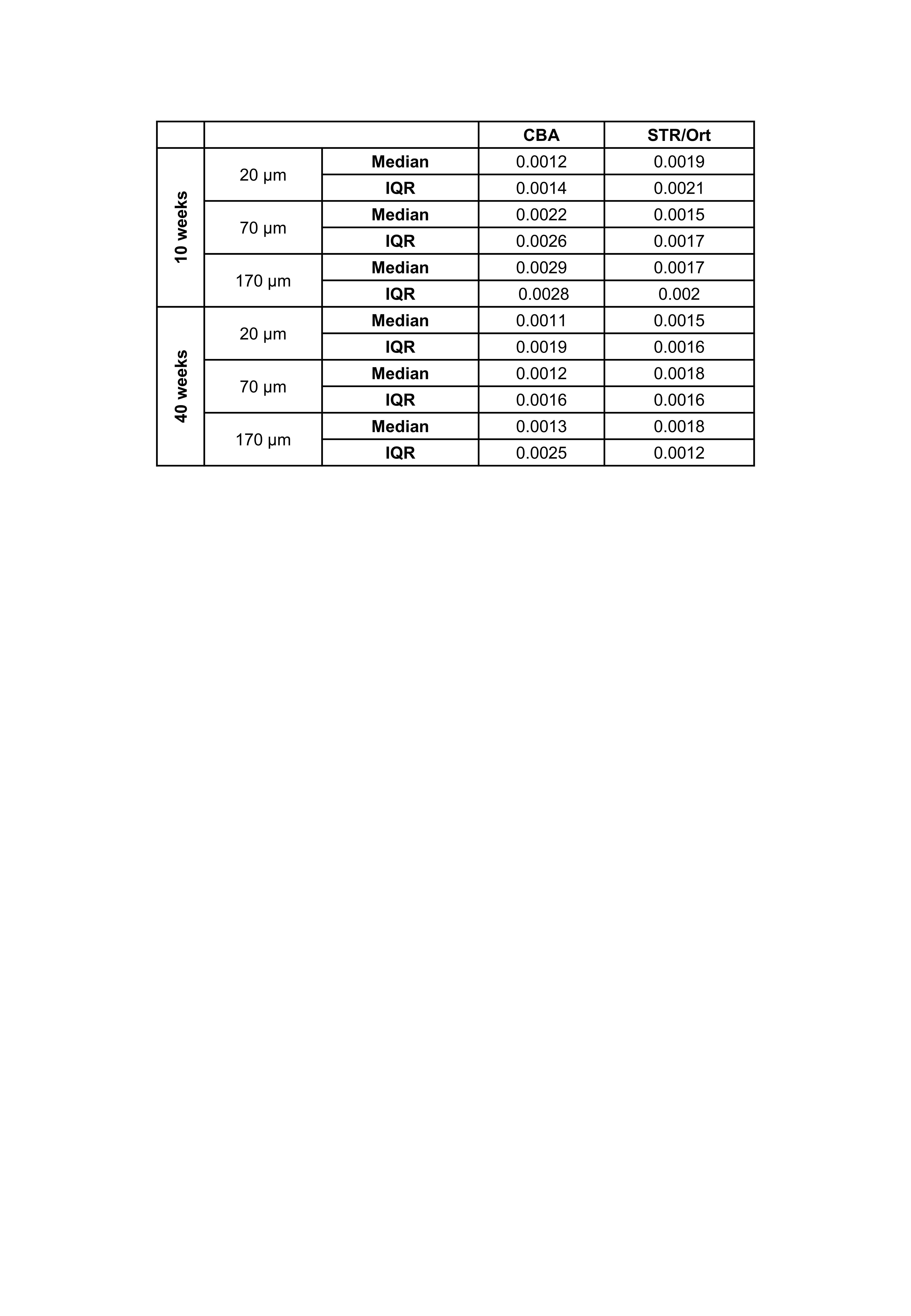

Supplement: Supplementary file 2 — Supporting File 2: advs76716‐sup‐0002‐TableS1‐S3.zip. [file ADVS-9999-e76716-s001.zip › Supplementary Table 2.TIF]

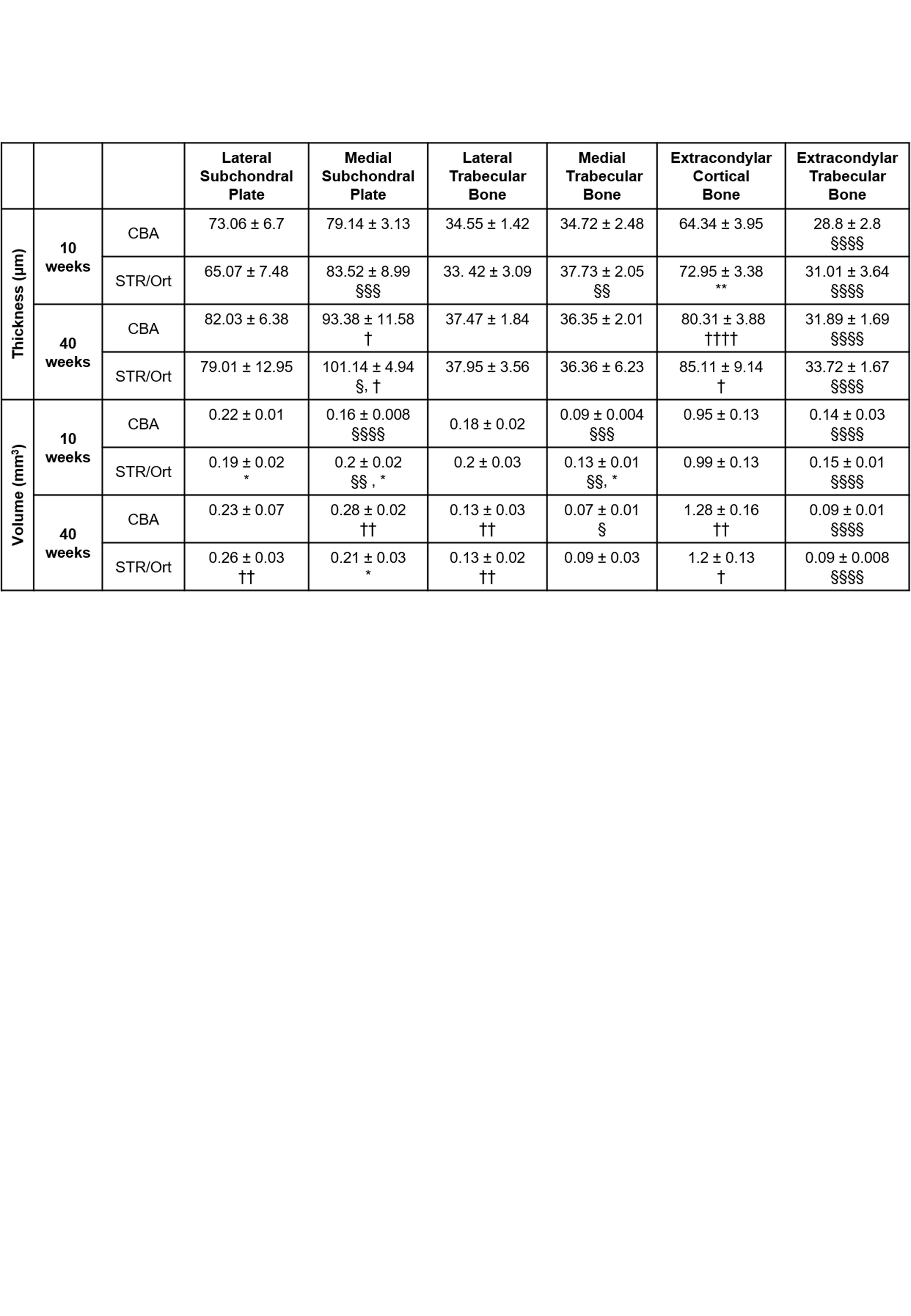

Supplement: Supplementary file 2 — Supporting File 2: advs76716‐sup‐0002‐TableS1‐S3.zip. [file ADVS-9999-e76716-s001.zip › Supplementary Table 3.TIF]
